# PD-1 deficiency reshapes the TCRβ repertoire and T-cell developmental programs associated with susceptibility to lupus-like autoimmunity

**DOI:** 10.3389/fimmu.2026.1911666

**Published:** 2026-09-15

**Authors:** Xianliang Hou, Zhenyu Liu, Guiya Lu, Shaofeng Jiang, Jinjun Qiu, Chunhong Li, Meng Meng, Zipeng Hu, Yinping Lei, Huilin Lao, Lemin Chen, Yue Wen, Siyan Xie, Yuqiang Liang

**Affiliations:** 1 Laboratory Center, Guangxi Key Laboratory of Metabolic Reprogramming and Intelligent Medical Engineering for Chronic Diseases, The Second Affiliated Hospital of Guilin Medical University, Guilin, China; 2 Department of Central Laboratory, Shenzhen Hospital (Longgang), Beijing University of Chinese Medicine, Shenzhen, China; 3 Department of Nuclear Medicine, Jinling Hospital, Affiliated Hospital of Medical School, Nanjing University, Nanjing, China; 4 College of Life Sciences, Guangxi Normal University, Guilin, China; 5 Guangxi Key Laboratory of Tumor Immunology and Microenvironmental Regulation, School of Basic Medicine Sciences, Guilin Medical University, Guilin, China; 6 Shenzhen Pingshan District People’s Hospital, Pingshan Hospital, Southern Medical University, Shenzhen, China

**Keywords:** autoimmune disease, high-throughput sequencing, immune repertoire sequencing, PD-1, systemic lupus erythematosus, T cell receptor

## Abstract

**Introduction:**

Programmed cell death protein-1 (PD-1) functions as a pivotal immune checkpoint, essential for maintaining peripheral immune tolerance, and serves as a significant therapeutic target in cancer immunotherapy. Nevertheless, the relationship between PD-1 deficiency and baseline alterations in T-cell receptor (TCR) repertoire organization, as well as the susceptibility to lupus-like autoimmunity, remains inadequately elucidated.

**Methods:**

In this study, we integrated immune repertoire sequencing with bulk RNA sequencing of peripheral blood mononuclear cells (PBMCs) to delineate the organization of the TCRβ repertoire and genotype-associated transcriptional changes in PD-1-deficient mice. To assess autoimmune susceptibility following an environmental immune challenge, we utilized a *Campylobacter jejuni* (CJ-S131)-induced lupus-like autoimmune model.

**Results:**

Our findings revealed that PD-1-deficient mice exhibited alterations in TCRβ repertoire diversity, complementarity-determining region 3 (CDR3) length distributions, TRBV/TRBJ segment usage, V-D-J recombination patterns, and clonotype abundance. Through transcriptomic analysis, we identified significant alterations in the expression of genes linked to lymphocyte development and the maintenance of the T-cell receptor (TCR) repertoire. Concurrently, our predicted regulatory-network analysis highlighted FOXC1, E2F1, GATA2, and MEF2A as potential upstream transcription factors implicated in subsets of these transcriptional changes. PD-1 deficiency was correlated with modified proportions of T cells, natural killer (NK) cells, B cells, and the CD4^+^CD25^+^CD127^low/−^ putative regulatory T-cell population. Additionally, PD-1-deficient splenocytes demonstrated heightened proliferative responses upon polyclonal stimulation. In the context of CJ-S131-induced autoimmune challenge, PD-1-deficient mice exhibited elevated levels of lupus-associated autoantibodies and more severe lupus-like renal pathology.

**Discussion:**

Collectively, our findings suggest that PD-1 deficiency is linked to disrupted TCRβ repertoire organization, extensive immune dysregulation, and an increased vulnerability to infection-induced lupus-like autoimmunity.

## Introduction

Programmed cell death protein 1 (PD-1), a constituent of the immunoglobulin superfamily, is a 55-kDa type I transmembrane protein (1). PD-1 serves as a pivotal inhibitory immune checkpoint, modulating T-cell activation and contributing to the preservation of peripheral immune tolerance. The therapeutic inhibition of the PD-1 pathway has emerged as a significant approach in cancer treatment, aimed at augmenting antitumor immune responses. Nevertheless, the disruption of PD-1 signaling can result in immune-related adverse events (irAEs), including the onset or worsening of autoimmune conditions such as systemic lupus erythematosus (SLE) (2). Clinical reports of novel systemic autoimmune disorders following anti-PD-1 therapy highlight the critical role of PD-1 signaling in sustaining immune tolerance (3, 4). Consequently, elucidating the immune alterations associated with compromised PD-1 signaling may offer mechanistic insights pertinent to the susceptibility to autoimmune complications subsequent to immune checkpoint blockade.

The organization of the T-cell receptor (TCR) repertoire is a crucial factor in antigen recognition and T-cell-mediated immunity. TCR diversity arises through V(D)J recombination during lymphocyte development, with the hypervariable complementarity-determining region 3 (CDR3) playing a pivotal role in determining antigen specificity. This region is formed through the recombination of V, D, and J gene segments, along with junctional nucleotide insertions and deletions (5–7). Beyond the initial generation of TCR sequences, the peripheral TCR repertoire is further refined by processes such as thymic selection, peripheral selection, clonal expansion, survival, and antigen-driven responses. SLE is a systemic autoimmune disorder characterized by a loss of immune tolerance, the production of pathogenic autoantibodies, and immune-mediated damage to multiple organs (8). Although increased expression of PD-1 has been observed in patients with established SLE, this upregulation is generally regarded as an adaptive feedback mechanism associated with chronic immune activation (9, 10). In contrast, the disruption of PD-1 signaling due to immune checkpoint blockade or genetic deficiency eliminates a crucial inhibitory pathway, potentially facilitating the breakdown of immune tolerance (2, 11). These observations underscore the existence of distinct immunological contexts. Nevertheless, the association between PD-1 deficiency and baseline modifications in TCR repertoire organization, as well as transcriptional programs pertinent to lymphocyte development and repertoire maintenance, remains inadequately elucidated.

Recent advancements in high-throughput sequencing have facilitated the detailed characterization of complex TCR repertoires (12). In this study, we employed immune repertoire sequencing (IR-seq) to analyze the TCRβ repertoire in PD-1^-/-^, PD-1^+/-^, and wild-type (WT) mice, with a focus on repertoire diversity, CDR3 length distributions, TRBV/TRBJ segment usage, V-D-J combination patterns, and clonotype abundance. Additionally, bulk PBMC RNA sequencing was conducted to investigate genotype-associated transcriptional alterations, including changes in genes and pathways related to lymphocyte development and TCR repertoire maintenance. To assess autoimmune susceptibility in response to an environmental immune challenge, we utilized a *Campylobacter jejuni* (CJ-S131)-induced lupus-like autoimmune model, which has been previously documented to elicit lupus-like autoimmune manifestations in susceptible hosts (13–15). This infection-induced model serves as a complementary framework for investigating the interplay between PD-1 deficiency and environmental immune stimulation. The overarching objective of this study was to ascertain whether PD-1 deficiency correlates with modifications in TCRβ repertoire organization, genotype-associated transcriptional alterations, and heightened susceptibility to infection-induced lupus-like autoimmunity.

## Methods

### Animals and ethical approval

PD-1**^-/-^** mice were generated by Cyagen Biosciences (www.cyagen.com) using the CRISPR/Cas9 technology. C57BL/6N mice, aged 6-8 weeks, were housed at the Animal Laboratory Center of Guilin Medical University under specific pathogen-free conditions, with a 12-hour light/dark cycle. Routine monitoring was conducted for body weight, growth, development, and behavior. A cohort of 75 mice, comprising wild-type (WT) C57BL/6N mice, PD-1^-/-^ mice, and PD-1^+/-^ mice, was utilized for RNA sequencing (RNA-seq), immune repertoire sequencing (IR-seq), flow cytometry, MTT proliferation assays, and the development of a lupus-like autoimmune model. The number of animals used in each experimental procedure is detailed in the respective sections below. All animal experiments were performed in compliance with the institutional guidelines of Guilin Medical University for animal research and received approval from the Animal Ethics Committee of Guilin Medical University (approval no. GLMC202307038).

### RNA extraction, library construction, immune repertoire sequencing, and bioinformatics analysis

Peripheral blood mononuclear cells (PBMCs) were isolated from peripheral blood via Ficoll-Hypaque density-gradient centrifugation. Total RNA was extracted from PBMCs using TRIzol reagent (Ambion, Austin, TX, USA) following the manufacturer’s protocol. RNA purity was evaluated using a NanoPhotometer^®^ spectrophotometer (IMPLEN, CA, USA). The integrity of RNA was evaluated utilizing the RNA Nano 6000 Assay Kit in conjunction with an Agilent 2100 Bioanalyzer system (Agilent Technologies, CA, USA). Concurrently, RNA concentration was ascertained through the Qubit^®^ RNA Assay Kit on a Qubit^®^ 2.0 Fluorometer (Life Technologies, CA, USA). Subsequently, multiplex polymerase chain reaction (PCR) was employed to amplify the rearranged TCRβ CDR3 regions, following methodologies previously documented in the literature (6, 16). The resultant PCR products underwent purification via the QIAquick PCR Purification Kit. The final libraries were quantified using the Agilent 2100 Bioanalyzer.

These libraries were then subjected to cluster generation on a cBot system and sequenced in paired-end mode on an Illumina HiSeq 2000 platform. Sequencing errors and biases introduced during PCR amplification were rectified using MiTCR software (17). Additional platform-specific algorithms were employed to correct for PCR and sequencing errors within the Illumina sequencing data, as detailed in prior studies (18). Post-filtering, the paired-end reads were amalgamated into single contiguous sequences. Sequence alignment was conducted using the MiTCR program (MiLaboratory; https://github.com/milaboratory/mitcr). After alignment, the frequencies of each distinct DNA sequence, amino acid sequence, and V-J combination were calculated. TCR repertoire diversity was assessed using the D50 index, richness, the Simpson index, the Shannon index, and the inverse Simpson index (19).

### RNA sequencing and bioinformatics analysis

Three biological replicates were prepared for RNA sequencing for each group, three wild-type C57BL/6N mice, three PD-1^-/-^ mice, and three PD-1^+/-^ mice. Both male and female mice were randomly included in the study without stratification by sex. All nine samples were submitted for sequencing simultaneously and processed within the same submission batch. Total RNA was extracted using TRIzol reagent (Ambion, Austin, TX, USA) and quantified with a NanoDrop 2000 spectrophotometer. Libraries were constructed using the NEBNext Ultra RNA Library Prep Kit for Illumina, and their concentrations were quantified post-purification. Sequencing was performed in paired-end mode on an Illumina HiSeq 2000 platform, and read quality was assessed using FastQC (version 0.11.9) (20). Low-quality reads were removed, and the remaining reads were aligned to the GRCm38 (mm10) reference genome using HISAT2 version 2.2.1. StringTie (version 2.2.1) was used to process the aligned reads and assemble the transcripts. PCA was performed independently of differential-expression analysis using the global TPM expression matrix after expression filtering. Genes with a summed FPKM greater than 0.5 across all samples were retained, and PCA was performed using the R princomp() function with cor = TRUE, which standardizes the variables based on the correlation matrix. Gene expression counts were quantified utilizing HTSeq (version 0.13.5). Differential gene expression across the genotype groups was assessed by pairwise comparisons using the DESeq2 R package (version 1.34.0). The normalization of gene expression counts was conducted using the median-of-ratios method as implemented in DESeq2. P-values were adjusted via the Benjamini-Hochberg procedure to control the false discovery rate. Differentially expressed genes (DEGs) were identified based on criteria of |log2(fold change)| > 1.0 and an adjusted *p-*value < 0.05.

The clusterProfiler R package (version 3.1) was employed to conduct KEGG and GO enrichment analyses of the DEGs, with *p*-values < 0.05 considered statistically significant for these enrichment analyses. To investigate potential upstream transcriptional regulatory associations, NetworkAnalyst was utilized to construct a predicted transcription factor (TF)-target gene regulatory network based on curated interactions from JASPAR. The constructed network was visualized to identify candidate transcription factors that may be associated with the observed transcriptional changes. These predicted associations are considered hypothesis-generating and should not be interpreted as evidence of direct transcriptional regulation or causality.

### Flow cytometry

Spleen cells were collected and processed according to previously described methods (21). The proportions of T cells, B cells, and NK cells were determined using the following antibodies: CD45-PerCP-Cy5.5, CD3-FITC, CD4-PE-Cy7, CD8-APC-Cy7, CD19-APC, and NK1.1-PE. The percentage of CD4^+^CD25^+^CD127^low/-^ putative regulatory T-cell population was identified using CD3-FITC, CD4-PE-Cy7, CD25-PE, CD45-PerCP-Cy5.5, and CD127-APC, as previously described (22, 23). A CD4^+^CD25^+^CD127^low/-^ surface-marker strategy was used to identify this putative population without intracellular staining. Because intracellular Foxp3 staining was not performed, the identified CD4^+^CD25^+^CD127^low/-^ population should be interpreted strictly as a putative regulatory T-cell population rather than as definitively identified Foxp3^+^ regulatory T cells (22–24). Unstained cells were included to assess background autofluorescence and assist in establishing baseline fluorescence signals. Single-stained controls for each fluorochrome were used to calculate the compensation matrix and correct for spectral overlap in the multicolor panel. All anti-mouse antibodies were purchased from URIT Medical Electronic Co., Ltd.

### MTT proliferation assay and serum autoantibody testing

Lymphocyte proliferation was assessed using an MTT assay. Freshly isolated splenocytes were seeded in 96-well flat-bottom tissue culture plates at a density of 2.5×10^6^ cells/mL in 200 µL of RPMI-1640 medium supplemented with 10% fetal calf serum (FCS). For nonspecific polyclonal stimulation, cells were incubated with phytohemagglutinin (PHA), while control wells received culture medium alone. After 72 hours of incubation, 10 µL of MTT solution (5 mg/mL) was added to each well. Following an additional 4-6 hours of incubation, the resulting formazan crystals were solubilized using 150 µL of dissolving solution. Optical density (OD) was measured at 540 nm using a microplate reader. Lymphocyte proliferative responsiveness was determined by the difference in OD values between PHA-stimulated and unstimulated cultures. Serum concentrations of ANA, anti-dsDNA, anti-ssDNA, and anti-snRNP/Sm antibodies were measured using specific enzyme-linked immunosorbent assay (ELISA) kits (Shanghai Enzyme-linked Biotechnology Co., Ltd., China) in accordance with the manufacturer’s guidelines.

### CJ-S131-induced lupus-like autoimmune model

A cohort of 24 mice, aged 6 to 8 weeks, was randomly selected, comprising six PD-1^-/-^ mice, six PD-1^+/-^ mice, and twelve WT mice. The groups were matched for sex, age, and body weight. The mice were distributed into four groups (n = 6 per group), each consisting of three females and three males. Three groups-PD-1^-/-^, PD-1^+/-^, and WT mice-underwent the *Campylobacter jejuni* strain S131 (CJ-S_131_) induction protocol to assess genotype-associated differences in susceptibility to infection-triggered lupus-like autoimmunity. The CJ-S131-immunized WT mice were designated as the WS group, serving as the infection-induced WT reference group, thereby facilitating comparisons of lupus-like autoimmune manifestations across genotypes subjected to the same induction protocol. An additional group of six WT mice received saline treatment and served as the normal control (NC) group. Subsequent to the reactivation of CJ-S_131_, formaldehyde-inactivated bacteria were prepared using 0.5% sterile formaldehyde and subsequently combined with an equivalent volume of Freund’s Complete Adjuvant (FCA) to create a stable emulsion. On day 1, 50 µL of the bacterial suspension was injected subcutaneously into the neck region of mice in the three experimental groups, while 50 µL of sterile saline was administered subcutaneously to mice in the NC group. On day 15, 200 µL of the bacterial suspension was administered via the tail vein to mice in the experimental groups, while 200 µL of sterile saline was administered via the tail vein to mice in the NC group.

### Kidney tissues preparation and histological analysis

At the conclusion of the study, euthanasia was conducted using an overdose of sodium pentobarbital (150 mg/kg, administered intraperitoneally), followed by the immediate collection of renal tissues. All animals were provided with humane care in compliance with protocols sanctioned by the Animal Ethics Committee of Guilin Medical University (Approval no. GLMC202307038), and all procedures adhered to pertinent legal and ethical standards. Following three washes with chilled phosphate-buffered saline (PBS), the tissues were promptly fixed in 10% formaldehyde solution and subsequently embedded in paraffin. Paraffin-embedded tissues samples were sectioned to a thickness of 5 µm, subjected to baking at 60 °C, deparaffinized, and subsequently stained with hematoxylin and eosin (H&E). More than five microscopic fields from each kidney section were examined using a light microscope to evaluate renal histopathological alterations. A semiquantitative histopathological scoring system was utilized to quantitatively assess the severity of renal pathological changes. The H&E-stained kidney sections were assessed in a blinded manner by an independent pathologist who was not informed of the experimental group assignments. Renal histopathological changes were graded on a scale from 0 to 4 based on the severity of glomerular lesions, as follows: Grade 0, normal histology; Grade 1, mild focal mesangial cell proliferation; Grade 2, extensive and diffuse mesangial cell proliferation; Grade 3, severe mesangial cell proliferation with lobular formation and basement membrane thickening; and Grade 4, severe glomerular crescent formation, glomerulosclerosis, necrosis, and tubular atrophy or epithelial shedding. When lesions of different grades coexisted in the same kidney section, the score was assigned based on the most severe lesion observed. Group scores were subsequently summarized and subjected to statistical analysis.

### Statistical analyses

Unless otherwise specified, data are presented as mean ± standard deviation (SD) values or as percentages (%). The experimental unit was the individual animal. Precise biological sample sizes (n) are detailed in the respective Methods sections and figure legends. The normality of the distribution of continuous data was evaluated prior to conducting parametric tests, where applicable. Parametric tests were employed for data conforming to the requisite distributional assumptions, while nonparametric tests were utilized when these assumptions were violated. For comparisons between two independent groups, an unpaired t-test or an appropriate nonparametric test was conducted. Analyses involving multiple groups were performed using one-way or two-way ANOVA, or mixed-effects analysis as appropriate, followed by the specified multiple-comparison procedure for each analysis, such as Tukey’s or Dunnett’s multiple-comparison test, where applicable. Wilcoxon tests were employed for the relevant nonparametric comparisons, as specified. The statistical test and *post hoc* procedure applied to each analysis are indicated in the corresponding figure legends.

Animals that perished prior to the predetermined experimental endpoint were excluded from analyses necessitating samples or measurements at that specific endpoint, and no attempts were made to impute missing biological data. The study included both male and female mice, which were combined within experimental groups; sex was not considered as an independent variable in the statistical models. For RNA-seq differential expression analyses, P-values were adjusted using the Benjamini-Hochberg method to control the false discovery rate, with statistical significance defined as an adjusted P-value of less than 0.05 in conjunction with an absolute log2(fold change) greater than 1.0. For other statistical analyses, a two-sided P-value of less than 0.05 was deemed statistically significant unless stated otherwise. Statistical analyses were conducted using SPSS version 20.0, alongside specific statistical software as required for individual analyses.

## Results

We employed IR-seq to characterize the TCRβ CDR3 repertoires of PD-1-deficient (PD-1^-/-^, and PD-1^+/-^) mice and WT mice. On average, 20.48 million paired-end reads per sample were obtained across the nine samples using the Illumina sequencing platform. Low-quality reads were removed according to previously described quality-control criteria. On average, 5.63% (range, 4.96-6.58%) of reads were filtered out using this procedure. An average of 12,473 clonotypes was identified from the sequencing data, with mean numbers of 3,205, 2,381, and 2,074 unique nucleotide sequences per sample in the PD-1^-/-^ group, PD-1^+/-^ group, and WT group, respectively, after removal of redundant identical sequences within each sample. Nonproductive out-of-frame TCR sequences constituted a subset of each repertoire. The proportion of these sequences varied among samples, ranging from 4.47% to 7.74%, with an average of 6.27%. A comprehensive description of the distribution of reads and clonotypes is provided in **Supplementary Table S1**. For the purposes of the present analysis, out-of-frame TCRβ sequences were operationally categorized as the “pre-selection” repertoire, whereas in-frame sequences were categorized as the “post-selection” repertoire (25, 26). These terms are used solely to distinguish sequence categories based on their productive status. Because these sequences were obtained from peripheral blood rather than directly from thymocyte developmental subsets, the peripheral out-of-frame repertoire should not be interpreted as a direct representation of the primary thymic pre-selection V(D)J recombination landscape. Rather, in this study, it represents a peripheral nonproductive sequence compartment that is less directly influenced by antigen receptor-mediated functional selection than the productive in-frame repertoire.

### Altered CDR3 length and diversity in PD-1-deficient mice

Initially, we characterized the comprehensive TCRβ repertoire within PBMCs from the PD-1-deficient mice and conducted a comparative analysis with WT mice. Our analysis revealed the presence of multiple expanded clonotypes at the overall repertoire level across all samples, amid a backdrop of numerous less expanded clonotypes (**Figures 1A, B**). To evaluate the diversity of the TCRβ CDR3 repertoire among the three groups, we employed the D50 index, Shannon index, Simpson index, and richness index (**Supplementary Figure S1**). Elevated values of the D50 index, Shannon index, and richness index are indicative of enhanced repertoire diversity, whereas a reduced Simpson index corresponds to increased diversity. Both PD-1^-/-^ and PD-1^+/-^ groups demonstrated numerically higher TCRβ CDR3 diversity compared to WT mice; however, these differences did not reach statistical significance.

**Figure 1:**
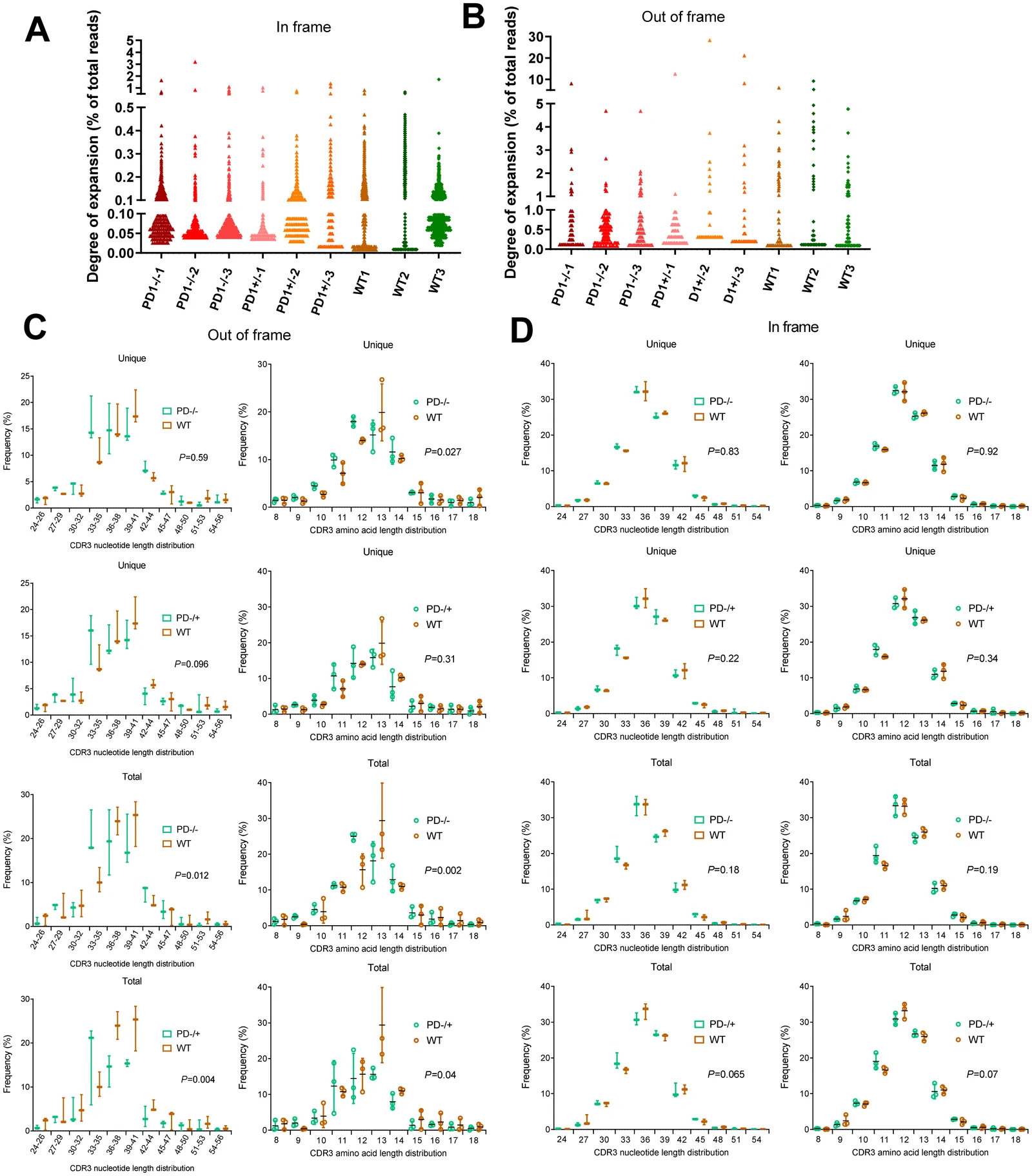
Degree of clonal expansion and altered TCRβ CDR3 length distributions in PD-1-deficient and wild-type (WT) mice. **(A, B)** Scatterplots of all recovered clones from each sample in the **(A)** in-frame and **(B)** out-of-frame repertoires. Each dot represents an individual clone, and clonal size is expressed as a percentage of the total T-cell receptor (TCR) repertoire. **(C, D)** Comparison of TCRβ CDR3 nucleotide and amino acid lengths between PD-1-deficient and WT mice. Analyses were performed on both the total TCRβ repertoire (incorporating clonotype size) and unique (non-redundant) TCRβ clonotypes within the **(C)** out-of-frame and **(D)** in-frame repertoires. PD-1-deficient mice exhibited a shift toward shorter TCRβ CDR3 length distributions, characterized by increased frequencies of short CDR3 sequences and reduced frequencies of long sequences. Immune repertoire sequencing (IR-seq) data were derived from n = 3 biological replicates per group. Statistical significance was assessed by mixed-effects two-way ANOVA, with mouse identity included as a random effect.

The CDR3 region of the TCRβ chain underwent sequencing and analysis, allowing for the determination of the length distributions of the CDR3 nucleotide and amino acid sequences (**Figures 1C, D**). Within the total out-of-frame TCRβ repertoire, PD-1-deficient mice exhibited significantly shorter CDR3 nucleotide and amino acid sequences relative to WT mice. Among unique out-of-frame TCRβ clonotypes, CDR3 lengths also tended to be shorter in PD-1-deficient mice, although the differences were not consistently statistically significant (**Figure 1C**). A similar tendency toward shorter TCRβ CDR3 sequences was observed in the in-frame repertoire of PD-1-deficient mice, although the differences were not statistically significant (**Figure 1D**). For the unique out-of-frame repertoire, TCRβ CDR3 amino acid lengths ranged from 1 to 39 aa. The five most frequent CDR3 lengths were 12 (17.95%), 13 (15.14%), 14 (11.58%), 11 (9.89%), and 10 (4.47%) aa in the PD-1^-/-^ group; 13 (15.82%), 12 (14.23%), 11 (10.71%), 14 (7.71%), and 10 (3.89%) aa in the PD-1^+/-^ group; and 13 (19.87%), 12 (14.00%), 14 (10.21%), 11 (7.10%), and 19 (3.30%) aa in the WT group. In the total out-of-frame repertoire, the TCRβ CDR3 amino acid length distribution peaked at 12 aa (24.97%) in the PD-1^-/-^ group and at 13 aa in both PD-1^+/-^ group (15.60%) and WT (29.38%) group.

### Skewed usage of TRBV gene subfamilies in PD-1-deficient mice

The utilization of TRBV segments exhibited significant differences between PD-1-deficient and WT mice in the in-frame TCRβ repertoire (**Figure 2**), with additional disparities noted in the out-of-frame repertoire (**Supplementary Figure S2**). In the comprehensive in-frame TCRβ nucleotide repertoire, TRBV1, TRBV12-2, and TRBV16 showed significantly higher usage in the PD-1**^-/-^** group, whereas TRBV1, TRBV13-3, and TRBV31 showed significantly higher usage in the PD-1**^+/-^** group (**Figure 2A**). Among unique in-frame TCRβ nucleotide clonotypes, TRBV12-2 and TRBV30 showed significantly higher usage in the PD-1**^-/-^** group, whereas TRBV1, TRBV13-3, TRBV31, and TRBV5 showed significantly higher usage in the PD-1**^+/-^** group (**Figure 2B**). Altered usage of TRBV12-2, TRBV1, TRBV13-3, and TRBV31 was observed across both the total and unique TCRβ nucleotide repertoires of PD-1-deficient mice. For the TRBJ gene subfamilies, TRBJ2-7 usage was significantly higher in PD-1-deficient mice. In the peripheral out-of-frame TCRβ repertoire, however, the overall usage patterns of TRBV and TRBJ gene subfamilies in PD-1-deficient mice were generally similar to those in WT mice, although significant differences were observed for individual features, including some insertion lengths (**Supplementary Figure S2**). V-D-J combination frequencies were also compared among the three groups. Multiple V-D-J combinations differed significantly in the PD-1**^-/-^** group and PD-1**^+/-^** group relative to WT mice in both the in-frame and out-of-frame TCRβ repertoires (**Figure 3**; **Supplementary Figures S3**, **S4**). More differential V-D-J combinations were identified in the in-frame repertoire than in the out-of-frame repertoire. A subset of the differential V-D-J combinations among PD-1**^-/-^**, PD-1**^+/-^**, and WT mice was shared between the total and unique TCRβ repertoires (**Figure 3C**). Several differential V-D-J combinations were also shared between the PD-1**^-/-^** versus WT and PD-1**^+/-^** versus WT comparisons (**Figure 3D**).

**Figure 2:**
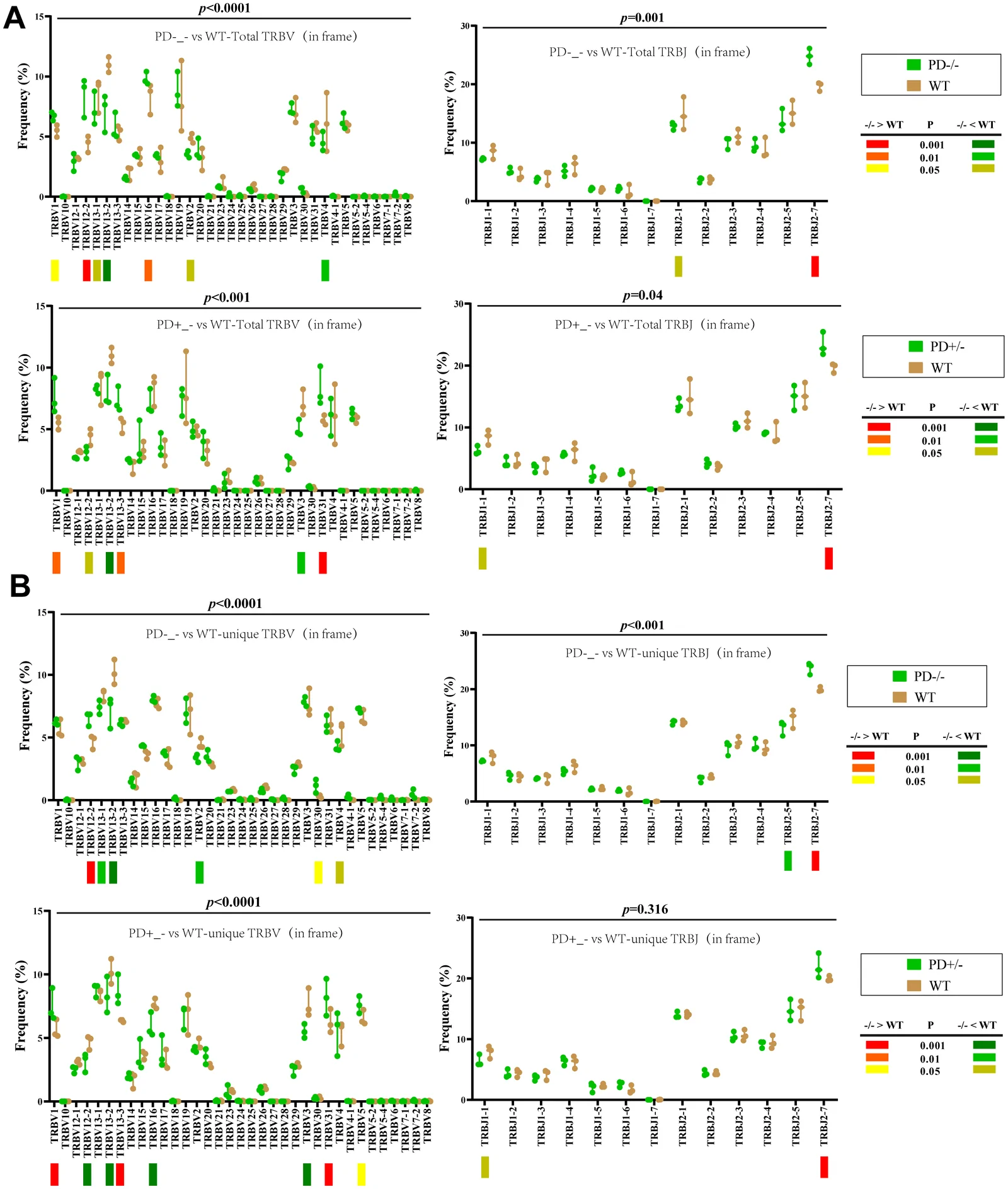
Differential usage frequency of TRBV and TRBJ gene segments in the in-frame repertoire of PD-1-deficient mice and WT mice. TRBV and TRBJ segment usage frequencies were compared among PD-1^-/-^, PD-1^+/-^ mice and WT mice across the total TCRβ nucleotide repertoires **(A)** and unique (non-redundant) TCRβ nucleotide repertoires **(B)**. Segments showing statistically significant differences among genotypes are highlighted in color. IR-seq data were derived from three biological replicates per genotype (n = 3 mice/group). Overall differences in segment usage among genotypes were assessed using mixed-effects two-way ANOVA.

**Figure 3:**
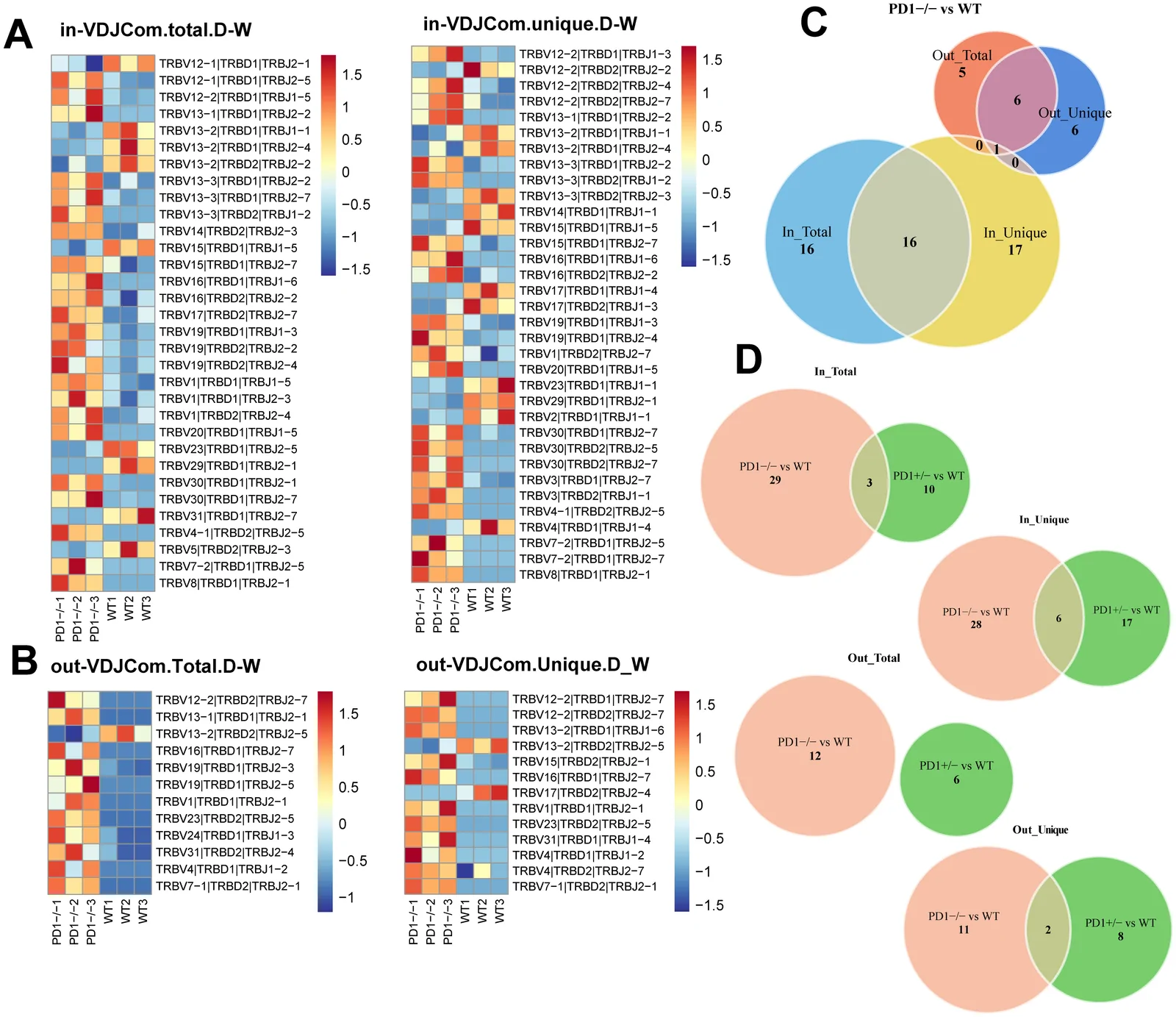
Differential utilization of V-D-J combinations in PD-1-deficient and wild-type (WT) mice is presented. **(A, B)** Heatmaps showing differential usage of V-D-J combinations between PD-1^-/-^ mice and WT mice in the in-frame **(A)** and out-of-frame **(B)** TCRβ repertoires. For each repertoire, analyses were performed using both the total TCRβ repertoire (weighted by clonotype abundance) and unique (non-redundant) TCRβ clonotypes. **(C, D)** Venn diagram displayed the overlap degree of the differential V-D-J combinations in PD-1^-/-^ mice among the out-of-frame total TCRβ repertoire, out-of-frame unique TCRβ repertoire, in-frame total TCRβ repertoire, and in-frame unique TCRβ repertoire **(C)**. Additionally, the Venn diagrams demonstrate the overlap in differentially utilized V-D-J combinations between PD-1^-/-^ vs WT and PD-1^+/-^ vs WT within the in-frame total TCRβ repertoire, in-frame unique TCRβ repertoire, out-of-frame total TCRβ repertoire, and out-of-frame unique TCRβ repertoire **(D)**. The numbers in the non-overlapping regions represent differential V-D-J combinations specific to each category, while those in the overlapping regions denote differential V-D-J combinations shared among the respective categories.

### Differential abundance of TCRβ CDR3 clonotypes in PD-1-deficient mice

We next compared the abundance of TCRβ CDR3 clonotypes among the three groups and identified multiple clonotypes with significantly different abundances among PD-1**^-/-^**, PD-1**^+/-^**, and WT groups (**Figure 4**). A greater number of differentially abundant CDR3 clonotypes was identified in the unique TCRβ repertoire than in the total TCRβ repertoire, and nearly all differentially abundant clonotypes identified in the total repertoire were also detected among those identified in the unique repertoire. For example, ASSAGQGDEQF, ASSGTGGYEQY, ASSLAGGNQDTQY, SAGTSEQY, and SARDRVSHEQY showed significantly higher abundance in the PD-1**^-/-^** group compared with the WT group, and ASSAGLGVDTQY and ASSRDIYAEQF showed significantly higher abundance in the PD-1**^+/-^** group compared with the WT group in both the total and unique TCRβ repertoires.

**Figure 4:**
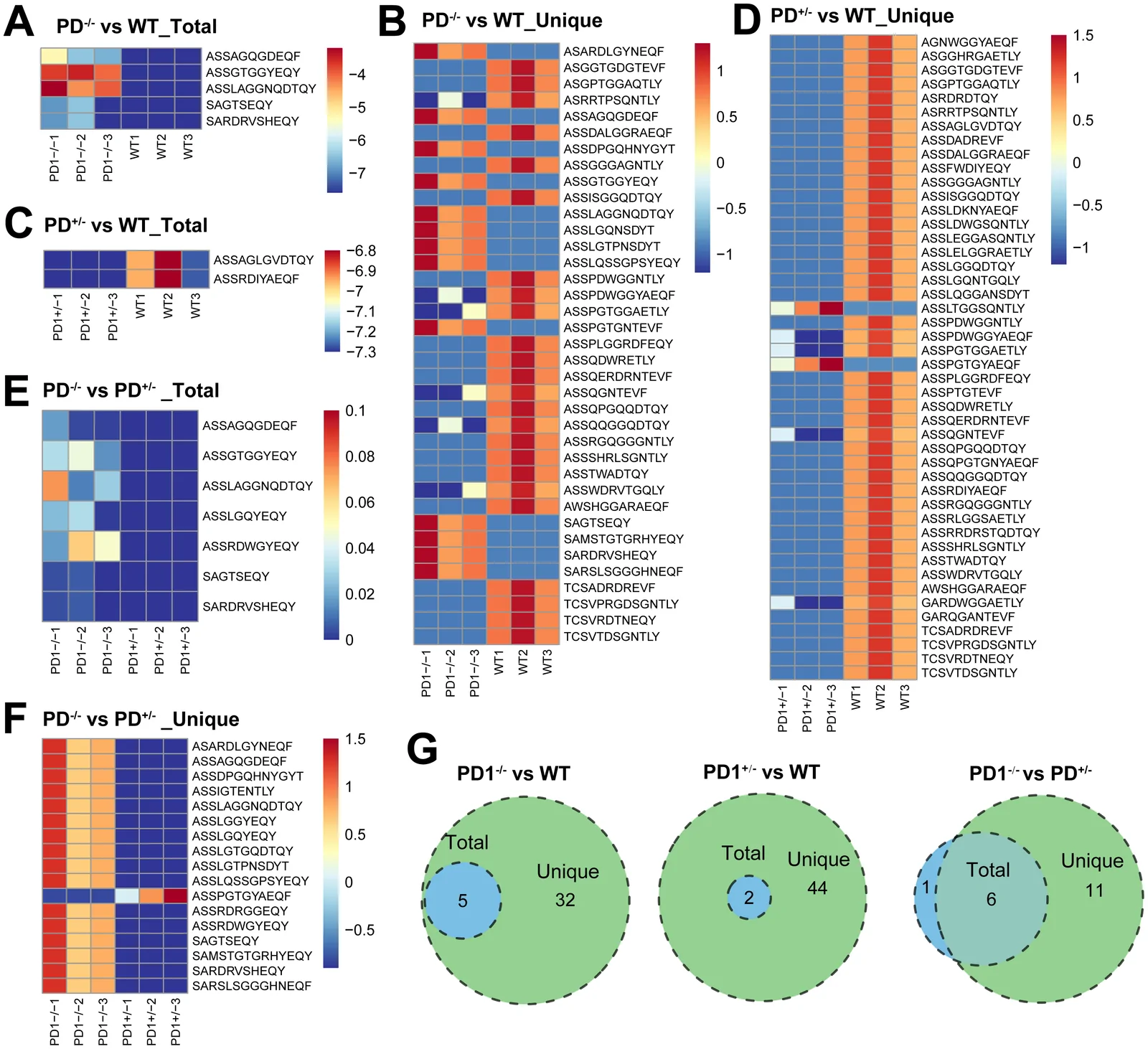
Differentially abundant TCRβ CDR3 amino acid sequences in PD-1-deficient mice. **(A–F)** Heatmaps display the differentially abundant in-frame TCRβ CDR3 sequences between PD-1^-/-^ mice and WT mice in the total TCRβ repertoire **(A)**, and unique TCRβ repertoire **(B)**, between PD-1^+/-^ mice and WT mice in the total TCRβ repertoire **(C)**, and unique TCRβ repertoire **(D)**, and between PD-1^-/-^ mice and PD-1^+/-^ mice in total TCRβ repertoire **(E)**, and unique TCRβ repertoire **(F)**. The Venn diagram displays the overlap degree of the differentially abundant in-frame TCRβ CDR3 sequences between the total TCRβ repertoire and unique TCRβ repertoire for the three comparisons: PD-1^-/-^ vs WT, PD-1^+/-^ vs WT, PD-1^-/-^ vs PD-1^+/-^ **(G)**. IR-seq data were derived from n = 3 biological replicates per group.

### Extensive genotype-associated transcriptomic alterations in PD-1-deficient mice

Compared with the WT group, the PD-1^-/-^ group exhibited 1,919 upregulated and 2,460 downregulated genes (adjusted *p* < 0.05 and |log2(fold change)| > 1.0) (**Figure 5A**). The comparison between the PD-1^+/-^ and WT groups identified 7,582 upregulated and 5,638 downregulated genes (**Figure 5B**), whereas the comparison between the PD-1^-/-^ group and PD-1^+/-^ groups identified 3,600 upregulated and 6,718 downregulated genes (**Figure 5C**). Given that these transcriptomic data were generated from bulk PBMCs, the extensive genotype-associated differences represent changes in the overall PBMC transcriptional landscape and may reflect both transcriptional alterations within immune-cell populations and differences in cellular composition among the three groups. The 15 most significantly upregulated genes and 15 most significantly downregulated genes are shown in heatmaps (**Figures 5D, E**; **Supplementary Figure S5**). Principal component analysis (PCA) was performed using the filtered global gene-expression dataset, with PC1 and PC2 accounting for 73.06% and 15.08% of the variance, respectively. The results showed that the PD-1^-/-^ group, PD-1^+/-^ group, and WT groups could be distinguished from each other in the PCA plot (**Figure 5F**).

**Figure 5:**
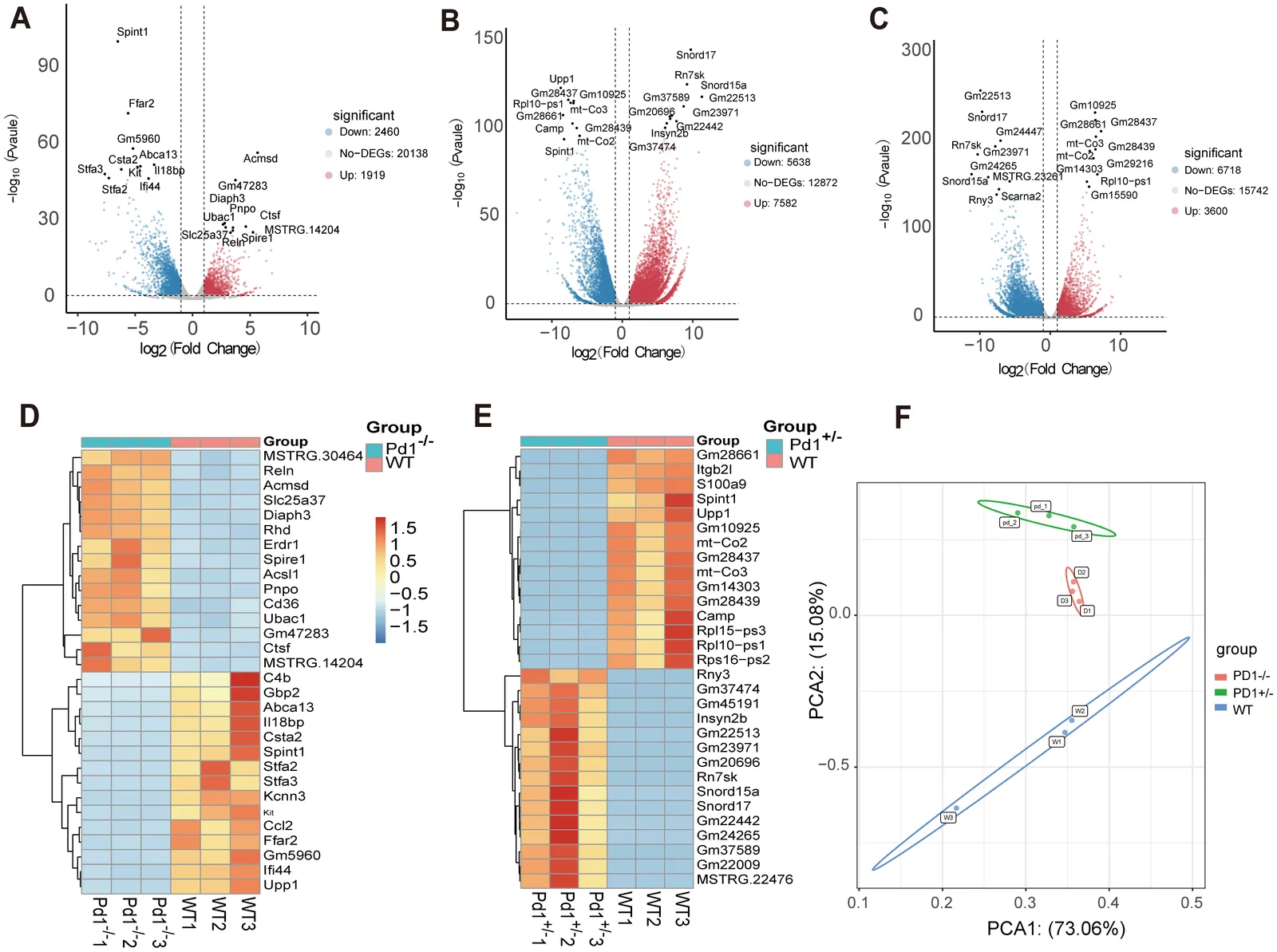
Transcriptional profiling and differentially expressed genes (DEGs) in PD-1-deficient mice. **(A–E)** Volcano plots **(A–C)** and heatmaps **(D, E)** illustrating the DEGs between the PD-1^-/-^ and WT groups **(A, D)**, the PD-1^+/-^ and WT groups **(B, E)**, and the PD-1^-/-^ and PD-1^+/-^ groups **(C)**. **(F)** Principal component analysis (PCA) based on the filtered global gene-expression dataset, illustrating sample-level transcriptional relationships among the PD-1^-/-^, PD-1^+/-^, and WT groups. PC1 and PC2 accounted for 73.06% and 15.08% of the variance, respectively. Transcriptomic data were derived from n = 3 biological replicates per group. DEGs were identified using DESeq2 based on a threshold of |log2(Fold Change)| > 1 and adjusted *p* < 0.05.

To characterize biological processes and pathways associated with the observed transcriptional differences, gene set enrichment analysis (GSEA) was performed. GSEA identified several enriched pathways between the PD-1^-/-^ and WT groups, including positive regulation of alpha/beta T-cell differentiation (NES = -1.74, *p* < 0.01), positive regulation of alpha/beta T cell activation (NES = -1.76, *p* < 0.01), recognition receptor signaling pathway (NES = -2.16, *p* < 0.01), thymic T cell selection (NES = 1.62, *p* < 0.05), regulation of T helper 1 cell differentiation (NES = -1.80, *p* < 0.01), T helper 2 cell differentiation (NES = -1.76, *p* < 0.01), B cell receptor signaling pathway (NES = 1.61, *p* < 0.01), and negative regulation of alpha/beta T cell proliferation (NES = -1.89, *p* < 0.01) (**Figure 6**; **Supplementary Figures S6**, **S7**). In addition, GO enrichment analysis further identified regulation of T-cell cytokine production, T-cell cytokine production, and T-cell differentiation involved in immune response as the three most highly enriched biological processes among DEGs between the PD-1^-/-^ group and the WT groups (**Supplementary Figure S8**). Protein-protein interaction (PPI) networks were constructed using the top 100 DEGs from the PD-1^-/-^ and PD-1**^+/-^** groups with the STRING database, and hub genes were subsequently identified using Cytoscape. The PPI network for the PD-1^-/-^ group comprised 98 nodes and identified 19 hub genes based on degree centrality, including *Akt1*, *Kit*, *Cdk1*, *Aurkb*, *Bub1*, *Aurka*, *Plk1*, *Melk*, *Mapk11*, *Met*, *Chek*, *Kif11*, *Kif4*, *Cenpe*, *Kif2c*, *Kif20a*, *Pbk*, *Ttk*, and *Chek1* (**Supplementary Figure S9A**). The PPI network for the PD-1^+/-^ group comprised 98 nodes and identified 24 hub genes based on degree centrality, including *Nedd8, Ubb, Rps27a, Ubc, Fau, Ccna2, Ccnb2, Ccnb1, Akt1, Mapk14, Kit, Cdk1, Aurkb, Cdk2, Bub1, Aurka, Plk1, Mapk3, Chek2, Grk3, Mtor, Akt2, Chek1, and Atm* (**Supplementary Figure S9B**).

**Figure 6:**
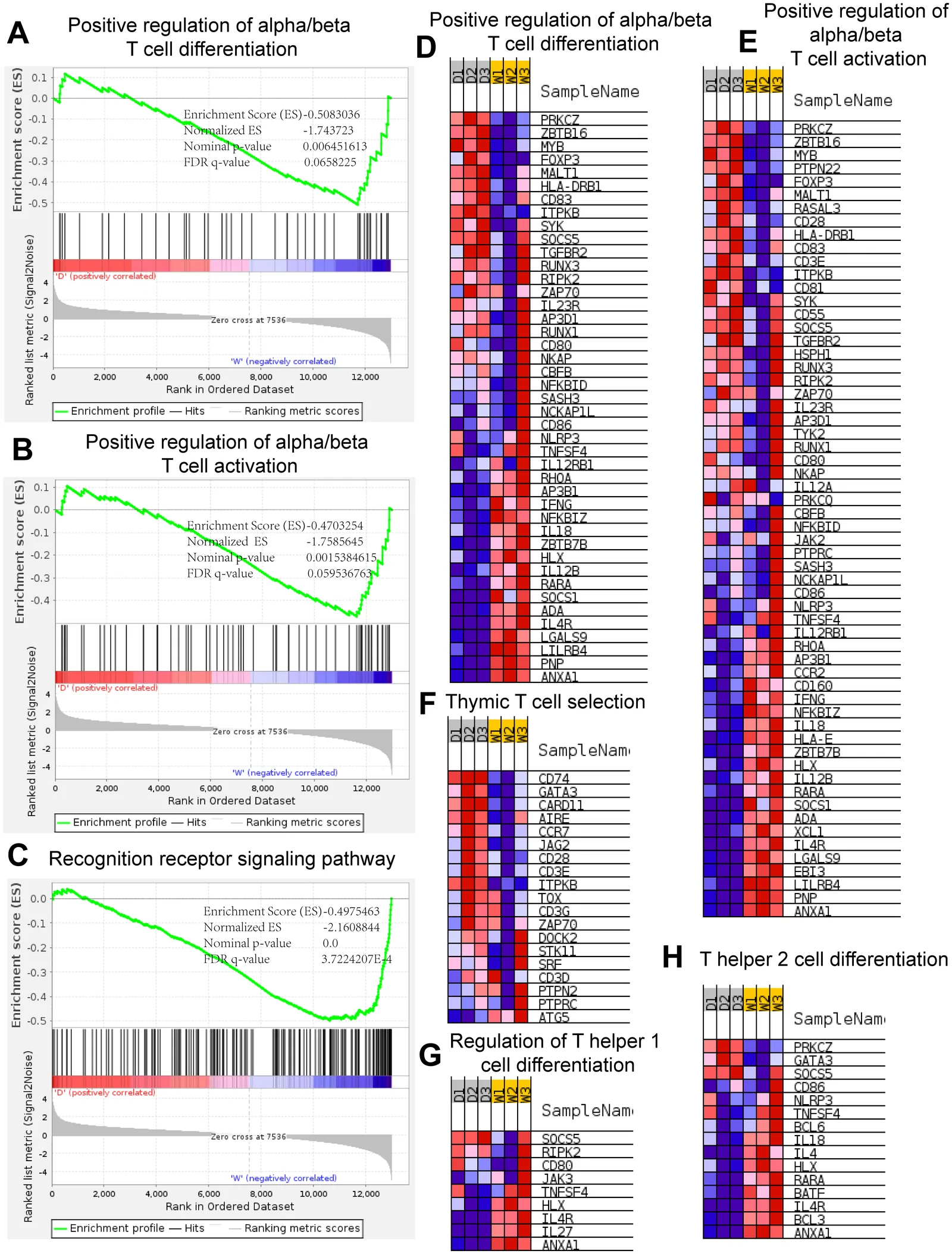
Gene Set Enrichment Analysis (GSEA) reveals altered immune-related signaling pathways in PD-1-deficient mice. **(A–C)** The GSEA plot showed that positive regulation of alpha/beta T cell differentiation, positive regulation of alpha/beta T cell activation, and recognition receptor signaling pathway were notably changed in PD-1^-/-^ mice. **(D–H)** Heatmaps showed the expression level of genes involved in the signaling pathways, including positive regulation of alpha/beta T cell differentiation, positive regulation of alpha/beta T cell activation, thymic T cell selection, regulation of T helper 1 cell differentiation, and T helper 2 cell differentiation, between PD-1**^-/-^** group and WT group. Transcriptomic data were derived from n = 3 biological replicates per group. DEGs were identified using DESeq2 based on a threshold of |log2(Fold Change)| > 1.0 and adjusted *p* < 0.05.

In the bulk PBMC transcriptomic dataset, we examined the expression of genes associated with lymphocyte development and TCR repertoire maintenance. Compared with WT mice, RAG1, DNTT, and PRKDC were significantly upregulated in the PD-1^-/-^ group (**Figure 7A**), while *XRCC4* was downregulated in this group (**Figure 7B**). *PRKDC*, *RAG1*, *XRCC5*, *XRCC6*, and *DNTT* were significantly upregulated in the PD-1^+/-^ group relative to the WT group (**Figure 7C**). Overall, the expression levels of genes associated with lymphocyte development and antigen-receptor assembly/repertoire maintenance were altered in PD-1-deficient mice. To explore potential upstream regulatory associations, a predicted transcription factor-target gene network was constructed using the NetworkAnalyst platform based on curated interaction data from JASPAR. The resulting network identified FOXC1, E2F1, GATA2, and MEF2A as candidate transcription factors predicted to be associated with several of the differentially expressed genes, including RAG1, DNTT, PRKDC, XRCC5, and XRCC6 (**Figure 7D**). Several of these candidate transcription factors also exhibited altered expression in PD-1-deficient mice compared with WT mice (**Figure 7E**). These findings identify FOXC1, E2F1, GATA2, and MEF2A as candidate transcription factors associated with the observed transcriptional alterations and warrant further functional validation. However, these data do not establish direct transcriptional regulation or causality.

**Figure 7:**
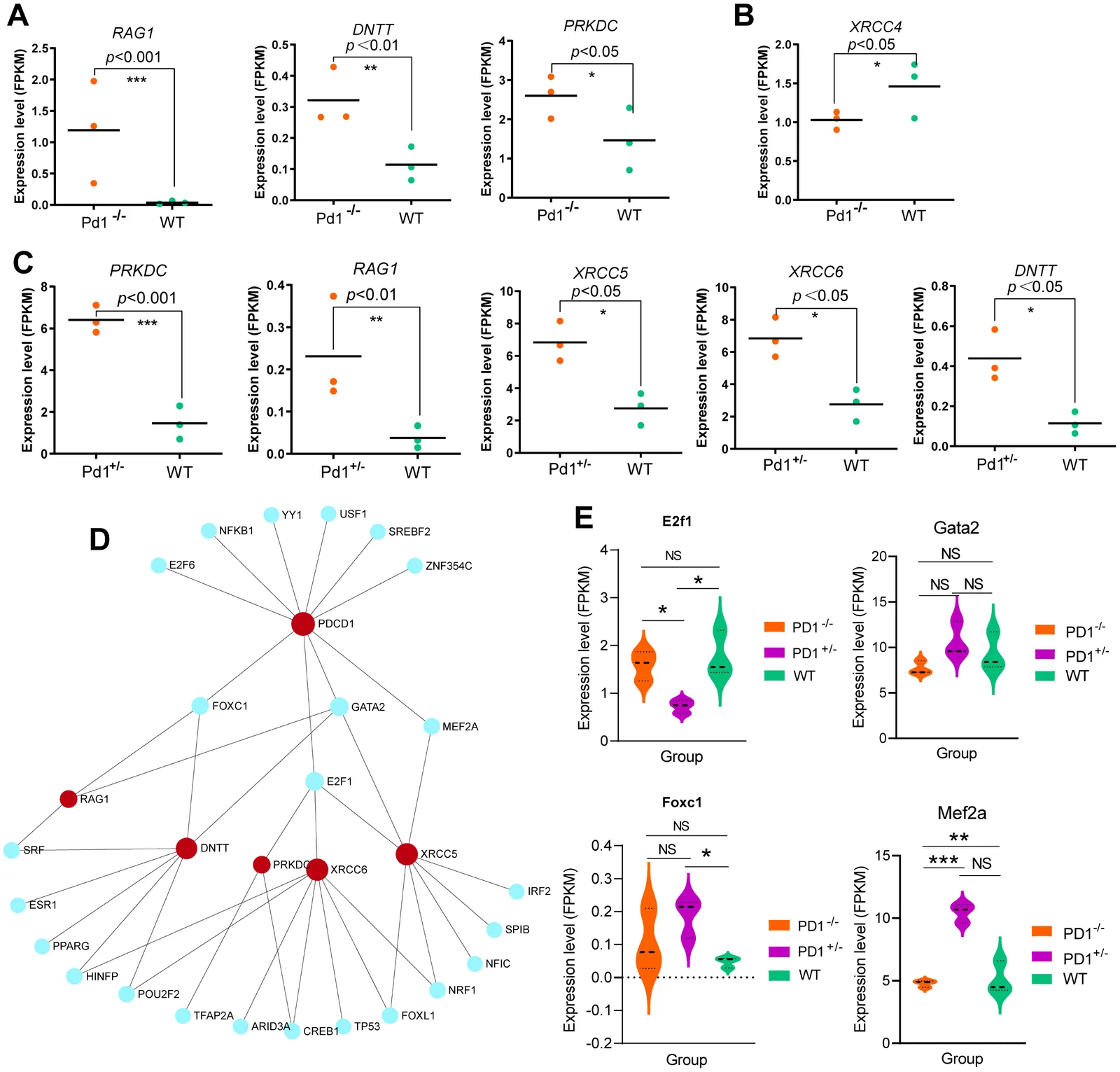
Differential expression of genes associated with T-cell development and TCR repertoire maintenance in PD-1-deficient mice. **(A, B)** Significantly upregulated **(A)** and downregulated **(B)** genes associated with T-cell development and TCR repertoire maintenance in PD-1^-/-^ group compared with WT group. Highly expressed genes associated with T-cell development and TCR repertoire maintenance in PD-1^+/-^ group compared with WT group **(C)**. Predicted transcription factor-target gene regulatory network generated using NetworkAnalyst based on curated JASPAR interactions, identifying FOXC1, E2F1, GATA2, and MEF2A as candidate upstream transcription factors associated with selected differentially expressed genes. The network represents computationally predicted regulatory associations rather than experimentally validated interactions. **(D)**. Comparison of FOXC1, E2F1, GATA2, and MEF2A expression between PD-1-deficient and WT mice. **(E)**. Data are representative of 3 replicates per group. For **(E)**, statistical significance was determined using a one-way ANOVA followed by Tukey’s multiple comparisons test. Significance is defined as *p < 0.05 and NS indicates not significant.

### Altered Immune cell composition and proliferative responsiveness in PD-1-deficient mice

We used flow cytometry to analyze immune-cell composition in the peripheral blood and spleen of mice from each group (**Supplementary Figures S10**, **S11**). Compared with WT mice, PD-1^-/-^ mice exhibited significantly higher proportions of T cells, NK cells, CD4^+^ T cells, and CD8^+^ T cells together with significantly lower proportions of B cells and the CD4^+^CD25^+^CD127^low/-^ putative regulatory T-cell population (**Figure 8B**). In the MTT assay, PD-1^-/-^ and PD-1^+/-^ splenocytes exhibited significantly greater proliferative responsiveness than WT splenocytes under nonspecific polyclonal PHA stimulation (p < 0.001) (**Figure 8C**).

**Figure 8:**
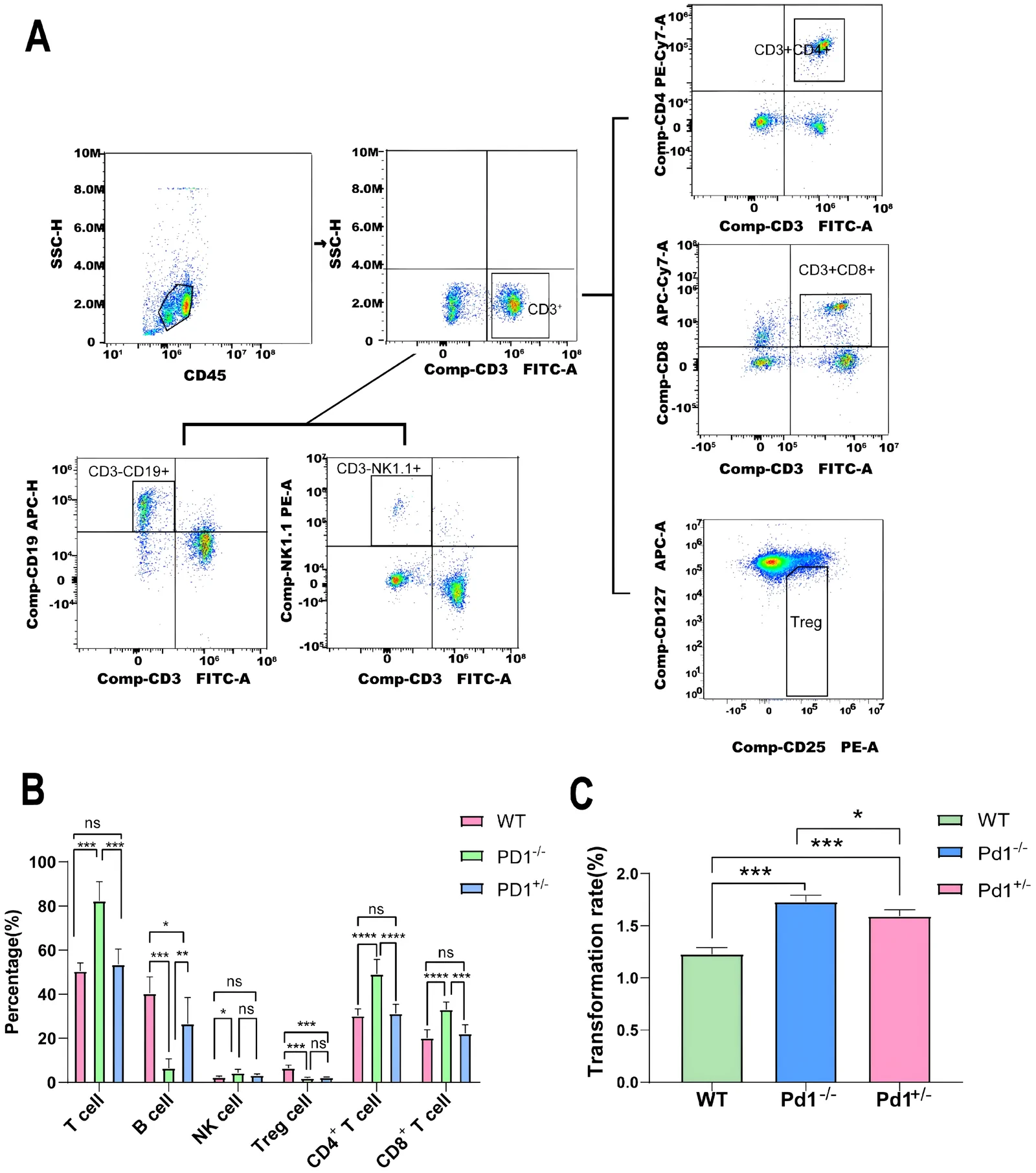
Altered immune cell composition and proliferative responses in PD-1-deficient mice. **(A)** The flow cytometry gating strategy for B cells and T cells, CD4^+^ T cells, CD8^+^ T cells, NK cells and CD4^+^CD25^+^CD127^low/-^ putative regulatory T-cell population. **(B)** Comparison of the percentage of T cells, CD4^+^ T cells, CD8^+^ T cells, B cells, NK cells, and CD4^+^CD25^+^CD127^low/-^ putative regulatory T-cell population among PD-1**^-/-^** group, PD-1**^+/-^** group and WT group. **(C)** Comparison of splenic lymphocyte proliferative responsiveness under PHA stimulation among PD-1**^-/-^** mice, PD-1**^+/-^** mice and WT mice. Significance is indicated by asterisks where * is *p* < 0.05, ** is *p* < 0.01, *** is *p* < 0.001. Data are presented as mean ± SD. The exact sample sizes are n = 6 for the WT group, n = 6 for the PD-1^+/-^ group, and n = 6 for the PD-1^-/-^ group. Statistical significance was determined using one-way ANOVA followed by Tukey’s multiple comparisons test.

### Increased lupus-associated autoantibodies and lupus-like renal pathological alterations in PD-1-deficient mice following CJ-S131 induction

We used formaldehyde-inactivated *Campylobacter jejuni* (CJ-S131) to establish an infection-triggered lupus-like autoimmune model in PD-1^-/-^, PD-1^+/-,^and WT mice and subsequently measured serum autoantibody levels and assessed renal histopathological alterations. Compared with the normal control (NC) group, WS mice exhibited increased levels of anti-ssDNA (*p* < 0.001), anti-snRNP/Sm (*p* < 0.01), ANA, and anti-dsDNA antibodies, with statistically significant increases in anti-ssDNA and anti-snRNP/Sm, supporting the induction of a lupus-like autoimmune phenotype (**Figure 9A**). Several autoimmune phenotypes showed a graded pattern across WS, PD-1^+/-^, and PD-1^-/-^ mice, although this pattern was not statistically significant across all comparisons and therefore should not be interpreted as a uniform gene-dosage relationship. The levels of anti-dsDNA (*p* < 0.001), ANA (*p* < 0.001), and anti-snRNP/SM (*p* < 0.001) were significantly higher in PD-1^-/-^ mice than in WS mice. Although the levels of these autoantibodies were also numerically higher in PD-1^+/-^ mice than in WS mice, the differences were not statistically significant. Renal histological examination revealed mesangial matrix expansion and glomerular hypertrophy in CJ-S131-immunized mice, with occasional inflammatory-cell infiltration (**Figures 9B–E**). H&E staining demonstrated structural renal pathological alterations but cannot establish glomerular immune-complex deposition. Together with the elevated circulating lupus-associated autoantibodies, these findings support the presence of renal pathology associated with lupus-like autoimmunity. Renal pathological alterations of varying severity were observed in all CJ-S131-immunized groups, whereas the NC group showed the lowest histopathological scores. The renal histopathological scores exhibited an apparent graded pattern across the groups (PD-1^-/-^ > PD-1^+/-^ > WS > NC), although this pattern should be interpreted descriptively rather than as evidence of a uniform gene-dosage effect. As shown in the quantitative scoring (**Figure 9F**), PD-1^-/-^ mice exhibited significantly higher renal histopathological scores than WS mice (*p* < 0.01).

**Figure 9:**
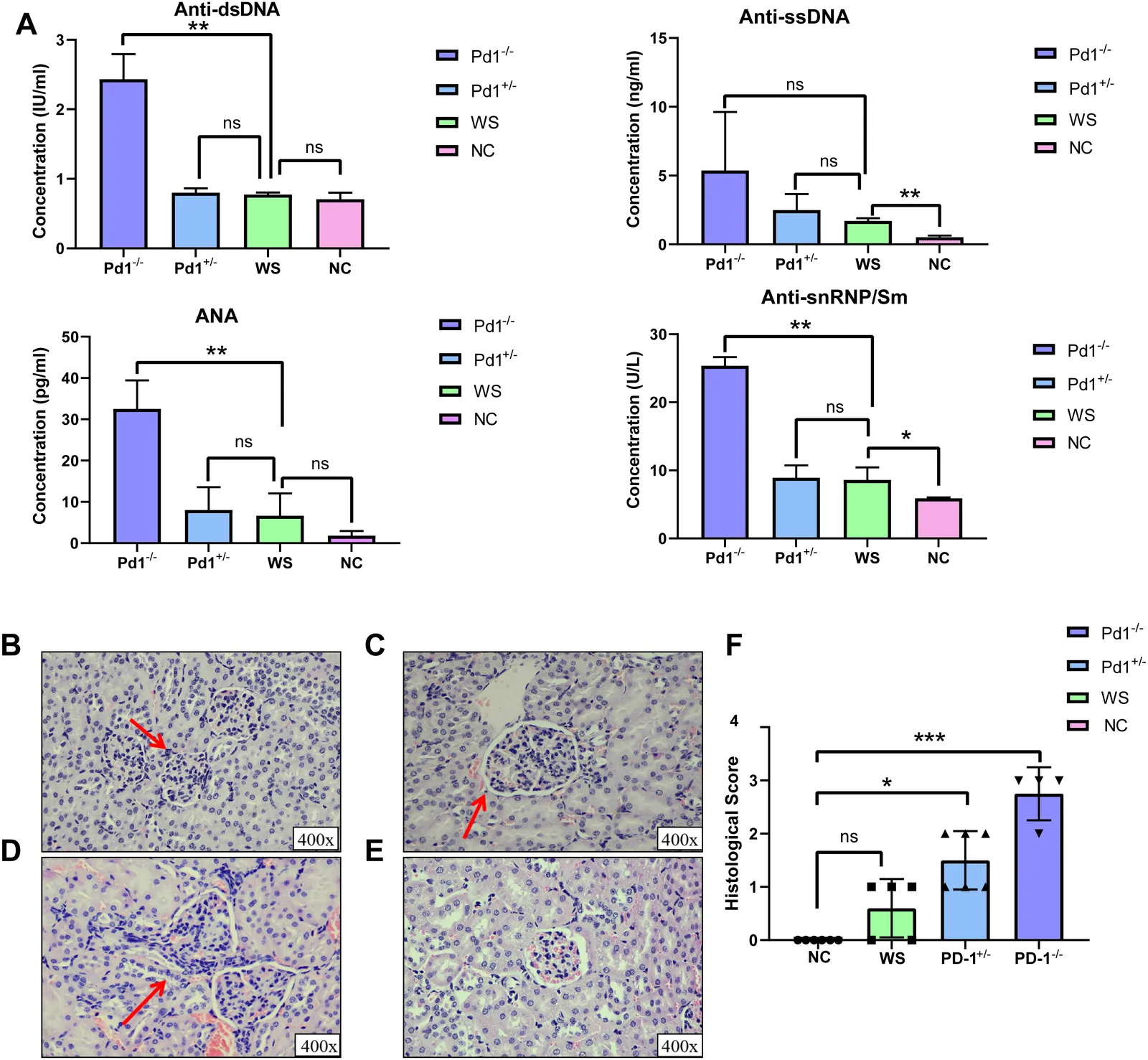
Elevated lupus-associated autoantibody levels and lupus-like renal pathological alterations in PD-1-deficient mice following CJ-S131 induction. **(A)** Comparison of the levels of anti-dsDNA, anti-ssDNA, ANA, and anti-snRNP/SM among PD-1^+/-^, PD-1^-/-^, WS, and NC group. **(B–E)** Representative Hematoxylin and Eosin (H&E) staining of kidney sections from mice treated with formaldehyde-inactivated *Campylobacter jejuni*. Red arrowheads indicate representative renal pathological alterations, including glomerular enlargement **(B)**, interstitial inflammatory cell infiltration **(C)**, and glomerular hypertrophy **(D)**. **(E)** Representative kidney section from the NC group. Original magnification × 400. H&E staining demonstrates structural renal pathological alterations but does not directly assess immune complex deposition. **(F)** Quantitative histopathological scoring of the kidney sections across the respective groups. Data are presented as mean ± SD. Autoantibody (ELISA) data were derived from n = 4 (PD-1^-/-^), n = 6 (PD-1^+/-^), n = 5 (WS), and n = 6 (NC) biological replicates due to disease-induced mortality during the model construction. Statistical significance was determined using a one-way ANOVA followed by Dunnett’s multiple comparisons test. Significance is defined as *p < 0.05, ***p < 0.001, and ns indicates not significant.

## Discussion

Programmed cell death protein 1 (PD-1, also referred to CD279 and encoded by the *PDCD1* gene),is primarily expressed on activated T cells and serves as a crucial inhibitory receptor in T-cell-mediated immunity (27). During T-cell activation, PD-1 mitigates stimulatory signals transmitted by the TCR and CD28 through its interactions with the ligands PD-L1 and PD-L2 (28). The PD-1 signaling pathway has been implicated in immune dysregulation associated with systemic lupus erythematosus (SLE) (29). Nevertheless, the impact of PD-1 deficiency on TCR repertoire organization and related immune mechanisms in the context of lupus-like autoimmunity remains incompletely understood. In this study, we characterized the TCRβ CDR3 repertoire organization in PD-1-deficient mice and investigated its association with transcriptional and immunological changes, as well as susceptibility to infection-induced lupus-like autoimmunity.

Alterations in TCR repertoire composition, including variations in CDR3 length distribution and clonotype diversity, may influence antigen recognition and reflect differences in T-cell selection and clonal dynamics (30). In comparison to WT mice, PD-1^-/-^ and PD-1^+/-^ mice exhibited a numerically greater diversity in TCRβ CDR3 sequences, though these the differences did not statistical significance. A critical consideration in interpreting these results is that both in-frame and out-of-frame TCRβ sequences analyzed in this study were derived from peripheral blood. In this study, the terms “post-selection” and “pre-selection” were operationally employed to differentiate between in-frame and out-of-frame sequence categories, respectively. Although nonproductive rearrangements are less directly influenced by antigen receptor-mediated functional selection compared to productive rearrangements, peripheral cells harboring these sequences have nonetheless undergone developmental and cellular selection processes at the host level. Consequently, the peripheral out-of-frame repertoire should not be construed as a direct representation of the primary thymic pre-selection V(D)J recombination landscape. Instead, the differences observed in these sequence categories offer complementary insights into the baseline peripheral TCR repertoire characteristics associated with PD-1 deficiency. PD-1-deficient mice demonstrated shorter TCRβ CDR3 sequences compared to WT mice, with the statistical significance of this difference varying across different repertoire categories. Previous research has shown that altered TCR repertoire characteristics, such as restricted diversity and modified CDR3 length distributions, are commonly observed in autoimmune diseases, including systemic lupus erythematosus (SLE), rheumatoid arthritis (RA), type 1 diabetes (T1D), and immunoglobulin A nephropathy (IgAN) (25, 31, 32). These findings suggest that changes in repertoire composition can accompany autoimmune immune dysregulation, although their functional significance is contingent upon the disease context and antigen specificity. Our prior experiments revealed that shorter CDR3 sequences were enriched during both thymic and antigen-driven selection, and were more prevalent in public T-cell responses (26). Recent studies have demonstrated that both CDR3 length and specific amino acid usage constrain the selection of MHC-specific TCRs, with TCRs possessing CDR3 regions longer than 13 amino acids being less favored by MHC restriction (33). Despite the observed differences in CDR3 length, the functional implications cannot be directly deduced from repertoire sequencing alone. In this study, PD-1 deficiency was linked to baseline modifications in TCR repertoire characteristics, including skewed TRBV gene usage, altered V-D-J recombination patterns, and variations in CDR3 length distributions. Notably, PD-1 is not regarded as a direct regulator of the TCR V(D)J recombination machinery or the initial generation of CDR3 (34). Instead, PD-1 regulates T-cell signaling thresholds, activation, survival, selection, and clonal dynamics, thereby contributing to peripheral immune tolerance (9). Consequently, the TCR repertoire observed in PD-1-deficient mice may reflect changes in immune selection, clonal expansion, survival, or persistence due to impaired PD-1-mediated regulation rather than direct modifications of the V(D)J recombination process (35). Given that antigen specificity was not directly assessed for the altered clonotypes identified in this study, these repertoire changes should not be construed as direct evidence of the expansion of autoreactive T-cell clones. In alignment with the repertoire findings, bulk PBMC RNA sequencing revealed altered expression of genes involved in lymphocyte development and antigen-receptor assembly/repertoire maintenance, including RAG1, DNTT, PRKDC, XRCC5, and XRCC6. These associations indicate genotype-related differences in the transcriptional landscape of bulk PBMCs and should not be construed as evidence that PD-1 directly regulates the V(D)J recombination machinery. Several immune and repertoire-associated phenotypes demonstrated a graded pattern across WT, PD-1^-/-^ and PD-1^+/-^ mice, which is broadly consistent with a gene-dosage effect. The PD-1 pathway is known to modulate T-cell signaling thresholds and limit autoreactive immune responses (34), and complete disruption of PD-1 has been linked to lupus-like systemic autoimmunity in experimental models (36). In several endpoints of the present study, PD-1^+/-^ mice displayed phenotypes intermediate between those of WT and PD-1^-/-^ mice, suggesting a potential graded relationship between the degree of PD-1 deficiency and specific immunological phenotypes. Nonetheless, this pattern was not consistently observed across all endpoints, and the transcriptomic findings, in particular, did not reveal a uniform linear relationship among the three genotypes. Consequently, the current data do not substantiate a universal PD-1 gene-dosage effect. Rather, the relationship between the degree of PD-1 deficiency and immune dysregulation appears to be dependent on phenotype and context (37).

To elucidate transcriptional alterations associated with PD-1 deficiency and explore biological pathways potentially related to T-cell development and repertoire organization, we conducted bulk PBMC RNA sequencing in PD-1-deficient and WT mice. We identified extensive genotype-associated transcriptional differences, with DEGs enriched in multiple immune-related pathways, including positive regulation of alpha/beta T-cell differentiation and activation, thymic T-cell selection, T helper 1 and T helper 2 cell differentiation, B-cell receptor signaling, and negative regulation of alpha/beta T-cell proliferation. These findings extend the established role of PD-1 in regulating T-cell activation by showing that PD-1 deficiency is associated with broader alterations in immune-related transcriptional programs. In particular, pathways related to T helper cell differentiation and collagen-activated tyrosine kinase receptor signaling were altered in the bulk PBMC transcriptomic dataset. However, because these data were derived from bulk PBMCs, the observed transcriptional differences may reflect both changes in gene expression within individual immune-cell populations and genotype-associated differences in PBMC composition. Accordingly, these findings should be interpreted as genotype-associated alterations in the overall immune transcriptional landscape rather than as evidence of a specific cell-intrinsic regulatory mechanism. Among the DEGs, several genes with established roles in lymphocyte development and antigen-receptor assembly, including RAG1, DNTT, XRCC4, PRKDC, XRCC5, and XRCC6, showed altered expression in PD-1-deficient mice compared with WT mice. Regulatory-network analysis further identified FOXC1, E2F1, GATA2, and MEF2A as candidate upstream transcription factors associated with subsets of these genes; however, these computationally predicted relationships do not establish direct transcriptional regulation or causality. RAG proteins initiate V(D)J recombination by recognizing recombination signal sequences and introducing DNA double-strand breaks, whereas components of the non-homologous end-joining machinery, including Ku70 (XRCC6), Ku80 (XRCC5), PRKDC, XRCC4, and DNA ligase IV, participate in subsequent DNA-end joining; DNTT contributes to junctional diversification during antigen-receptor assembly (38, 39). Although these genes have well-established roles in V(D)J recombination and antigen-receptor assembly, their altered expression in the present bulk transcriptomic dataset does not demonstrate that PD-1 directly regulates this machinery. Rather, our findings indicate an association between PD-1 deficiency and broader transcriptional alterations involving genes related to lymphocyte development and TCR repertoire maintenance. Similarly, the NetworkAnalyst-derived regulatory network should be considered hypothesis-generating rather than functionally validated. Future studies incorporating transcription-factor perturbation, promoter-binding assays, and direct functional measurements of V(D)J recombination would be necessary to determine whether the predicted regulatory associations have functional or causal significance.

An important limitation of the present transcriptomic analysis is that RNA sequencing was performed using bulk PBMCs with a limited number of biological replicates (n = 3 per genotype). Consequently, the observed differential gene expression cannot distinguish cell-intrinsic transcriptional alterations within specific immune-cell populations from differences in transcript abundance resulting from variation in cellular composition. This consideration is particularly relevant because our flow-cytometric analyses demonstrated genotype-associated differences in the proportions of T cells, B cells, NK cells, and the CD4^+^CD25^+^CD127^low/-^ putative regulatory T-cell population. Therefore, at least part of the transcriptional differences observed in bulk PBMCs may reflect differences in immune-cell composition rather than direct transcriptional effects of PD-1 deficiency within individual cell populations. This limitation is particularly important when interpreting developmentally regulated genes such as RAG1 and DNTT. Accordingly, their altered expression should be interpreted as being associated with changes in lymphocyte developmental programs and TCR repertoire maintenance rather than as evidence of direct PD-1-mediated transcriptional regulation. Moreover, the unexpectedly large number of DEGs observed in the PD-1^+/-^ versus WT comparison indicates that the transcriptomic response to partial PD-1 deficiency is not simply intermediate between WT and complete PD-1 deficiency. Therefore, the transcriptomic findings do not support a uniform linear gene-dosage relationship and may instead reflect complex genotype-associated alterations in immune-cell composition and cellular transcriptional states. Given the limited sample size and the potential contribution of genotype-associated differences in PBMC composition, these observations should be interpreted cautiously. Because both male and female mice were included without sex stratification, potential sex-related variation could not be independently evaluated and should also be considered when interpreting the bulk transcriptomic findings. Future studies using larger cohorts, purified immune-cell subsets, or single-cell transcriptomic approaches will be required to distinguish cell-intrinsic transcriptional effects from those attributable to cellular composition and to more fully evaluate potential sex-related effects.

We further examined whether PD-1 deficiency was associated with alterations in immune-cell composition and lymphocyte proliferative responsiveness. Previous studies have shown that PD-1 signaling can modulate T-cell proliferation and cytokine production under different experimental conditions (40, 41). A previous study by Nishimura et al. (36) showed that lymphocytes from PD-1^-/-^ mice exhibited augmented proliferative responses. Consistent with these observations, PD-1^-/-^ and PD-1^+/-^ splenocytes in the present study exhibited significantly greater proliferative responsiveness than WT splenocytes in the MTT assay under nonspecific polyclonal PHA stimulation. This finding should be interpreted as enhanced proliferative responsiveness under polyclonal stimulation rather than as evidence of increased antigen-specific or autoreactive T-cell proliferation. Flow-cytometric analysis further demonstrated significant genotype-associated differences in the proportions of T cells, B cells, NK cells, and the CD4^+^CD25^+^CD127^low/-^ putative regulatory T-cell population. These findings are consistent with previous evidence that PD-1 signaling contributes to the regulation of lymphocyte activation and immune-cell homeostasis (28, 42). PD-1 signaling has also been implicated in regulatory T-cell homeostasis, including changes in Foxp3^+^ Treg populations, as demonstrated by Francisco et al. (43). However, because intracellular Foxp3 staining was not performed in the present study, our CD4^+^CD25^+^CD127^low/-^ population should be interpreted only as a putative regulatory T-cell population and not as definitively identified Foxp3^+^ regulatory T cells.

Given that PD-1 deficiency was associated with altered TCR repertoire organization and substantial changes in immune-cell composition, we further investigated whether these alterations were accompanied by increased susceptibility to autoimmune activation following an environmental immune challenge. Indeed, following CJ-S131 induction, PD-1-deficient mice exhibited increased levels of lupus-associated autoantibodies, including anti-dsDNA, ANA, and anti-snRNP/Sm, together with more pronounced renal histopathological alterations compared with WS mice. Several autoimmune phenotypes showed an apparent graded pattern, with PD-1^-/-^ mice generally exhibiting the most pronounced abnormalities and PD-1^+/-^ mice showing intermediate changes for some endpoints. Although this pattern was broadly consistent with a graded effect of PD-1 deficiency, it was neither uniform nor statistically significant across all measurements and therefore does not establish a universal gene-dosage relationship. Taken together, these findings indicate that PD-1 deficiency is associated with increased susceptibility to infection-triggered lupus-like autoimmunity. These observations are consistent with previous reports showing spontaneous lupus-like autoimmune manifestations in PD-1-deficient C57BL/6 mice (27) and support the importance of intact PD-1 signaling in maintaining immune tolerance. Importantly, the role of PD-1 should be interpreted differently in primary, naturally occurring SLE and autoimmune manifestations associated with therapeutic PD-1 blockade. Our findings do not contradict previous reports describing increased PD-1 expression in patients with established SLE; rather, these observations reflect different stages and contexts of immune dysregulation. In established primary SLE, elevated PD-1 expression on T cells may represent an adaptive inhibitory response associated with chronic immune activation and persistent antigen stimulation (9, 10). Conversely, impairment of PD-1 signaling before or during autoimmune activation-whether through genetic deficiency or therapeutic checkpoint blockade-removes an important inhibitory pathway and may facilitate loss of immune tolerance in susceptible immunological contexts (11) (2). Accordingly, the baseline TCR repertoire and transcriptional alterations observed in PD-1-deficient mice may provide mechanistic insight relevant to susceptibility to PD-1 blockade-associated autoimmunity, rather than directly explaining the pathogenesis of primary SLE. This translational interpretation nevertheless requires caution. Germline PD-1 deficiency represents a lifelong absence of PD-1 signaling and therefore does not fully reproduce the temporal, pharmacological, and disease-context-specific effects of therapeutic PD-1 blockade in cancer patients. The present findings should therefore be regarded as providing mechanistic insight potentially relevant to susceptibility to immune checkpoint inhibitor-associated autoimmunity rather than as direct evidence of irAE development following therapeutic PD-1 blockade. Recent studies of immune checkpoint inhibitor-associated toxicities provide biological support for this interpretation. PD-1 blockade has been associated with changes in T-cell activation, differentiation, tissue distribution, and clonal dynamics, including the expansion and clonal evolution of pathogenic T-cell populations implicated in irAEs (36, 37). Single-cell and TCR-tracking studies have further demonstrated that such toxicities can involve the expansion and evolution of specific T-cell clones following checkpoint blockade (44). Similarly, Mulholland et al. reported that PD-1 blockade altered tissue T-cell composition and inflammatory activity, including increased T-cell accumulation and IFN-γ production (45). These findings support the concept that disruption of PD-1 signaling modifies T-cell states, selection, clonal dynamics, and inflammatory responses rather than directly altering antigen-receptor gene rearrangement. However, these findings from pharmacological PD-1 blockade should not be considered directly equivalent to the effects of germline PD-1 deficiency examined in the present study. Future longitudinal studies employing single-cell TCR sequencing before and after autoimmune induction could determine how individual T-cell clonotypes evolve during disease development and whether specific clonotypes undergo preferential expansion in the context of PD-1 deficiency.

We acknowledge several limitations of the present study. First, the histopathological evaluation of the kidneys was primarily conducted using H&E staining, which demonstrates structural lesions and inflammatory infiltration but cannot confirm glomerular immune-complex deposition (46). Future studies incorporating immunofluorescence staining for glomerular IgG and C3 deposition, together with functional renal assessments such as proteinuria or urinary albumin-to-creatinine ratio, would enable a more comprehensive characterization of renal pathology associated with lupus-like autoimmunity. Second, although intracellular Foxp3 staining is widely regarded as the gold standard for regulatory T-cell identification, this study employed a CD4^+^CD25^+^CD127^low/-^ surface-marker surrogate strategy (47–49). This strategy cannot definitively distinguish regulatory T cells from activated conventional CD4^+^ T cells, particularly under inflammatory conditions in which CD25 and CD127 expression may change. Therefore, findings related to this population should be interpreted with appropriate caution as representing a CD4^+^CD25^+^CD127^low/-^ putative regulatory T-cell population rather than definitively identified Foxp3^+^ Tregs. Future studies incorporating intracellular Foxp3 staining will be required for definitive identification and quantification of regulatory T cells. Third, the *in vitro* MTT proliferation assay employed PHA, a nonspecific polyclonal mitogen, and therefore reflects the proliferative responsiveness of splenic lymphocytes under polyclonal stimulation rather than antigen-specific T-cell activation or autoreactive T-cell expansion. Nevertheless, increased susceptibility to lupus-like autoimmunity *in vivo* was supported by complementary observations, including elevated lupus-associated autoantibody levels and renal histopathological alterations. Future pathogen-specific and autoantigen-specific stimulation assays could help determine whether PD-1 deficiency is associated with altered antigen-specific immune responses. Finally, the present CJ-S131-induced lupus-like autoimmune model did not include an FCA-only control group. Consequently, while the comparison between PD-1-deficient and WT mice subjected to the same CJ-S131 plus FCA induction protocol facilitates the evaluation of PD-1 deficiency effects under a controlled autoimmune challenge, it does not allow for the complete disentanglement of the individual contributions of bacterial antigen stimulation and nonspecific FCA-mediated inflammation. Future research incorporating FCA-only controls and antigen-specific stimulation methodologies will be instrumental in elucidating the distinct roles of microbial and inflammatory signals in PD-1 deficiency-associated lupus-like autoimmunity.

## Conclusion

In summary, PD-1 deficiency was linked to baseline modifications in TCRβ repertoire organization, immune cell composition, proliferative responsiveness, and transcriptional programs associated with lymphocyte development and repertoire maintenance. These modifications were accompanied by an increased susceptibility to infection-induced lupus-like autoimmunity, underscoring the critical role of intact PD-1 signaling in sustaining T-cell repertoire homeostasis and peripheral immune tolerance. The observed alterations in the TCRβ repertoire are more likely indicative of changes in immune selection and clonal dynamics rather than direct regulation of V(D)J recombination by PD-1. Collectively, these findings enhance our understanding of the immunological consequences of compromised PD-1 signaling and may have translational significance for elucidating susceptibility to autoimmune complications associated with therapeutic PD-1 checkpoint blockade. In this regard, further exploration of immune alterations associated with PD-1 deficiency may aid in identifying biomarkers for patients at heightened risk of irAEs (50) and guide the development of strategies that preserve antitumor immunity while minimizing autoimmune toxicity (51). Nonetheless, these potential translational implications necessitate validation within clinical cohorts undergoing PD-1 blockade therapy.

## Data Availability

The RNA-sequencing and immune repertoire sequencing datasets generated in this study have been deposited in the National Center for Biotechnology Information (NCBI) under BioProject accession number PRJNA1516105. Other data supporting the findings of this study are available from the corresponding author upon reasonable request.

## Supplementary material

Supplementary Figure 1 Diversity of the TCRβ repertoire in PD-1-deficient and WT mice. TCRβ CDR3 diversity in the in-frame repertoire (in-frame sequences) was estimated using the D50 index, Richness, Shannon index, and Simpson index. Immune repertoire sequencing (IR-seq) data were derived from n = 3 biological replicates per group. Statistical significance was determined using the Wilcoxon rank-sum test.

Supplementary Figure 2 Differential usage frequencies of TRBV and TRBJ genes in the out-of-frame repertoire of PD-1-deficient versus WT mice. Differential usage frequencies of TRBV and TRBJ gene segments between PD-1-deficient mice and WT mice across the total TCRβ nucleotide repertoires **(A)** and unique TCRβ nucleotide repertoires **(B)**. Overall differences between PD-1-deficient and WT mice were assessed using mixed-effects two-way ANOVA. TRBV and TRBJ gene segments showing statistically significant differences are highlighted in color. Data were derived from n = 3 biological replicates per group.

Supplementary Figure 3 The differential usage frequency of V-D-J combinations in PD-1^+/-^ mice compared with WT mice. **(A–D)** Heatmaps showing the differential V-D-J combinations between PD-1^+/-^ mice and WT mice in in-frame total TCRβ repertoire **(A)**, in-frame unique TCRβ repertoire **(B)**, out-of-frame total TCRβ repertoire **(C)**, out-of-frame unique TCRβ repertoire **(D)**. A Venn diagram showing the overlap of the differentially V-D-J combinations in PD-1^+/-^ mice among the out-of-frame total TCRβ repertoire, out-of-frame unique TCRβ repertoire, in-frame total TCRβ repertoire, and in-frame unique TCRβ repertoire **(E)**. The number of differential V-D-J combinations that were unique to each group was shown in the non-overlapping sections, and the number of differential V-D-J combinations that was common to two, three, and all four groups was indicated in the relevant overlapping areas. Data were derived from n = 3 biological replicates per group.

Supplementary Figure 4 Differential usage frequencies of V-D-J combinations between PD-1^-/-^ mice and PD-1^+/-^ mice. **(A–D)** Heatmaps showing differential V-D-J combinations between PD-1^-/-^ mice and PD-1^+/-^ mice in in-frame total TCRβ repertoire **(A)**, in-frame unique TCRβ repertoire **(B)**, out-of-frame total TCRβ repertoire **(C)**, out-of-frame unique TCRβ repertoire **(D)**. A Venn diagram showing the overlap degree of the differentially V-D-J combinations between PD-1^-/-^ mice and PD-1^+/-^ mice among the out-of-frame total TCRβ repertoire, out-of-frame unique TCR-β repertoire, in-frame total TCRβ repertoire, and in-frame unique TCRβ repertoire **(E)**. The number of differential V-D-J combinations that were unique to each group was shown in the non-overlapping sections, and the number of differential V-D-J combinations that was common to two, three, and all four groups was indicated in the relevant overlapping areas. Data were derived from n = 3 biological replicates per group.

Supplementary Figure 5 Heatmaps showing the differentially expressed genes (DEGs) between PD-1**^-/-^** group and PD-1**^+/-^** group. Transcriptomic data were derived from n = 3 biological replicates per group. DEGs were identified using DESeq2 based on a threshold of |log2(Fold Change)| > 1.0 and adjusted *p* < 0.05.

Supplementary Figure 6 Gene Set Enrichment Analysis (GSEA) reveals altered signaling pathways in PD-1^-/-^ mice. **(A–D)** GSEA plots showing enrichment of B cell receptor signaling pathway **(A)**, thymic T cell selection **(B)**, T helper 2 cell differentiation **(C)**, and negative regulation of alpha/beta T cell proliferation **(D)** were notably changed in PD-1^-/-^ mice. **(E)** Heatmaps showing the expression levels of genes involved in B cell receptor signaling pathway between PD-1**^-/-^** and WT groups. Transcriptomic data were derived from n = 3 biological replicates per group. DEGs were identified using DESeq2 based on a threshold of |log2(Fold Change)| > 1.0 and adjusted p < 0.05.

Supplementary Figure 7 Gene Set Enrichment Analysis (GSEA) showing additional altered signaling pathways in PD-1^-/-^ mice. **(A–F)** GSEA plots illustrating the enrichment of the Toll-like receptor signaling pathway **(A)**, cytokine-cytokine receptor interaction **(B)**, receptor inhibitor activity **(C)**, recognition receptor activity **(D)**, recognition receptor signaling pathway in response to virus **(E)**, and regulation of T helper 1 cell differentiation **(F)** in PD-1^-/-^ mice. Transcriptomic data were derived from n = 3 biological replicates per group.

Supplementary Figure 8 Gene Ontology (GO) and Kyoto Encyclopedia of Genes and Genomes (KEGG) enrichment analyses of differentially expressed genes (DEGs) in PD-1 gene-deficient mice. **(A, B)** GO enrichment analyses of the DEGs between PD-1**^-/-^** and WT mice **(A)** and between PD-1**^+/-^** and WT mice **(B)**, bar plots display the top 10 significantly enriched GO terms. **C**, **D** KEGG pathway enrichment analyses of the DEGs between PD-1**^-/-^** and WT mice **(C)**, between PD-1**^+/-^** and WT mice **(D)**, bubble plots dispaly the top 20 significantly enriched KEGG pathway. Transcriptomic data were derived from n = 3 biological replicates per group.

Supplementary Figure 9 PPI networks generated from the top 100 DEGs between PD-1**^-/-^** and WT mice**(A)**, and between PD-1**^+/-^**and WT mice **(B)**. Transcriptomic data were derived from n = 3 biological replicates per group.

Supplementary Figure 10 Representative flow cytometry plots of T-cell and B-cell populations. **(A)** Representative flow cytometry plots showing T cells populations in PD-1**^+/-^ (a)** and WT **(b)**, and PD-1**^-/-^** mice **(c)**. **(B)** Representative flow cytometry plots showing B cells populations in PD-1**^+/-^ (a)** and WT **(b)**, and PD-1**^-/-^** mice **(c)**. Flow cytometry data were derived from n = 6 biological replicates per group (3 females, 3 males). Statistical significance was determined using one-way ANOVA.

Supplementary Figure 11 Representative flow cytometry plots of NK-cell and putative regulatory T-cell populations. **(A)** Representative flow cytometry plots showing NK cells populations in PD-1**^+/-^ (a)** and WT **(b)**, and PD-1**^-/-^** mice **(c)**. **(B)** Representative flow cytometry plots showing the CD4^+^CD25^+^CD127^low/-^ putative regulatory T-cell population in PD-1**^+/-^ (a)** and WT **(b)**, and PD-1**^-/-^** mice **(c)**. The putative regulatory T-cell population was defined as CD3^+^ CD4^+^ CD25^+^ CD127^low/-^ cells. Flow cytometry data were derived from n = 6 biological replicates per group (3 females, 3 males). Statistical significance was determined using one-way ANOVA.

Supplementary Figure 12 Representative flow cytometry gating strategy for immune cell subset identification. Representative flow cytometry plots illustrating sequential gating for lymphocyte identification based on forward and side scatter characteristics, doublet exclusion, and the subsequent identification of T lymphocytes (CD3^+^), B lymphocytes (CD19^+^), natural killer cells (CD3^-^NK1.1^+^), CD4^+^ and CD8^+^ T-cell subsets, and the CD4^+^CD25^+^CD127^low/-^ putative regulatory T-cell population.

Supplementary Figure 13 Comprehensive sequential flow cytometry gating strategy for identifying the CD4^+^CD25^+^CD127^low^^/-^ putative regulatory T-cell population. This figure illustrates the sequential gating approach utilized to isolate the CD4^+^CD25^+^CD127^low/-^ putative regulatory T cell population. Representative flow cytometry plots are derived from the splenic suspension of a PD-1^+/-^ heterozygous mouse. The plots delineate the initial selection of the lymphocyte population, the subsequent exclusion of doublets to ensure singlet analysis, and the downstream identification of CD3^+^ T cells and the CD4^+^ T-cell subset. The final panel illustrates the gated CD4^+^CD25^+^CD127^low/−^ putative regulatory T-cell population within the CD3^+^CD4^+^ T-cell compartment.
