# Supplementary material for: PD-1 deficiency reshapes the TCRβ repertoire and T-cell developmental programs associated with susceptibility to lupus-like autoimmunity
Source: Front Immunol. 2026 Sep 15;17:1911666. doi: 10.3389/fimmu.2026.1911666 (PMC13619840; doi:10.3389/fimmu.2026.1911666)
Supplement: Supplementary file 1 [file DataSheet1.pdf]

Supplementary Figures

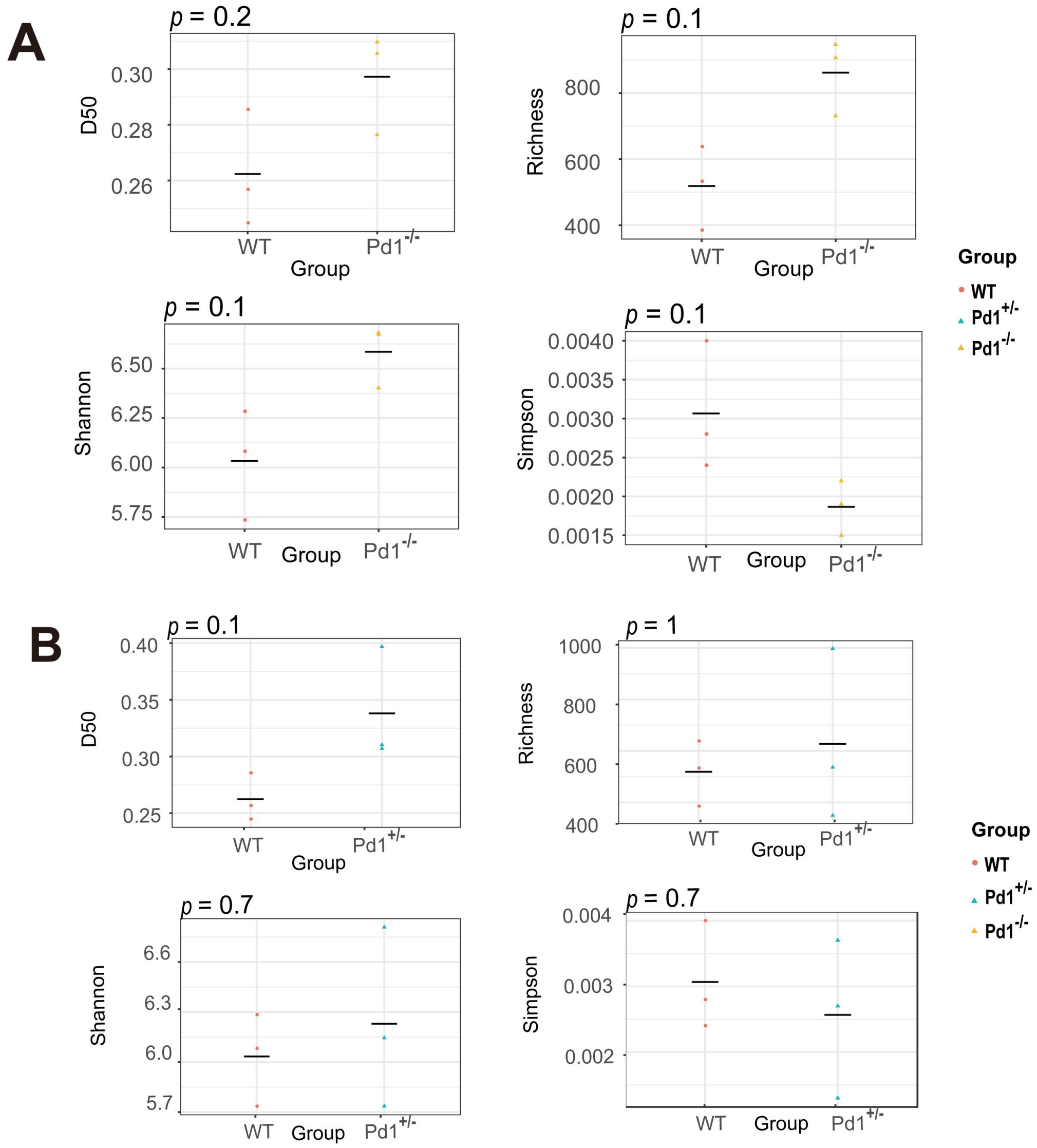

Figure S1. Diversity of the TCRβ repertoire in PD-1-deficient and WT mice. TCRβ CDR3 diversity in the post-selection repertoire (in-frame sequences) was estimated using the D50 index, Richness, Shannon index, and Simpson index. Immune repertoire sequencing (IR-seq) data were derived from n = 3 biological replicates per group. Statistical significance was determined using the Wilcoxon rank-sum test.

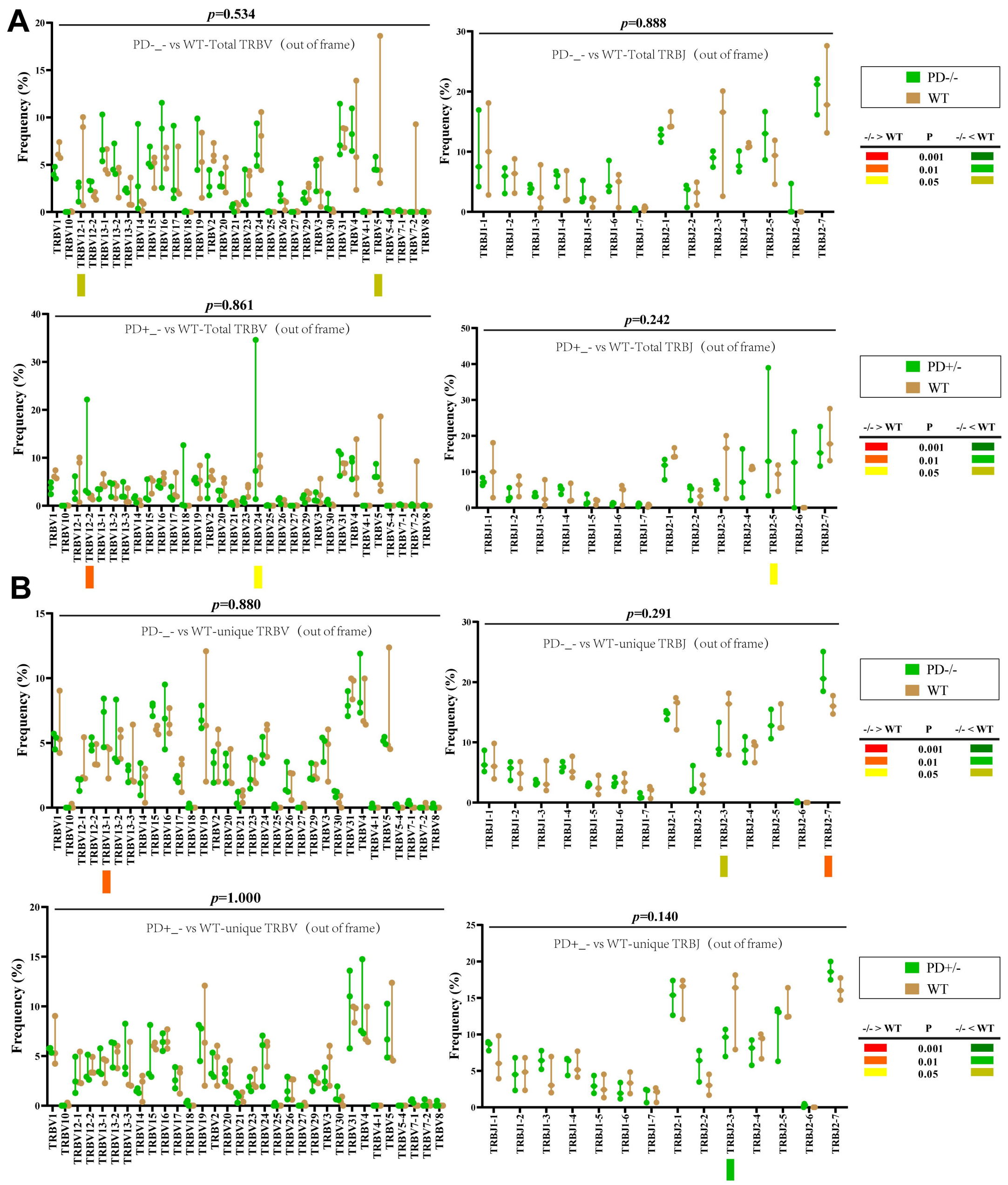

Figure S2. Differential usage frequencies of TRBV and TRBJ genes in the pre-selection repertoire of PD-1-deficient versus WT mice.Differential usage frequencies of TRBV and TRBJ gene segments between PD-1-deficient mice and WT mice across the total TCRβ nucleotide repertoires (**A**) and unique TCRβ nucleotide repertoires (**B**). The mixed effects two-way ANOVA was used to calculate for overall differences between PD-1-deficient mice and WT mice. TRBV and TRBJ gene segments showing statistically significant differences are highlighted in color. Data were derived from n = 3 biological replicates per group.

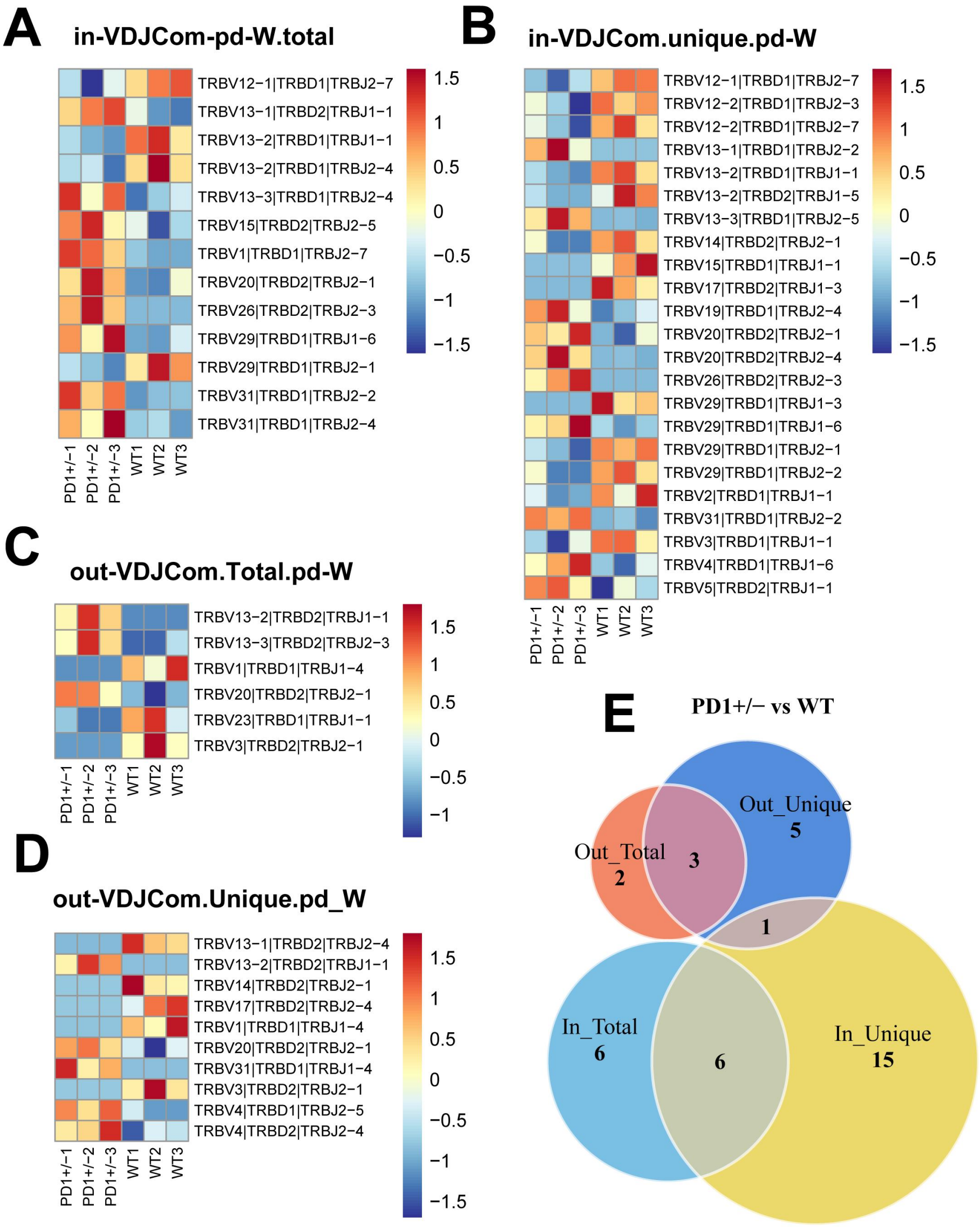

Figure S3. The differential usage frequency of V-D-J combinations in PD-1$^{+/-}$ mice compared to WT mice. **A-D** Heatmaps showing the differential V-D-J combinations between PD-1$^{+/-}$ mice and WT mice in post-selection total TCRβ repertoire (**A**), post-selection unique TCRβ repertoire (**B**), pre-selection total TCRβ repertoire (**C**), pre-selection unique TCRβ repertoire (**D**). A Venn diagram showing the overlap of the differentially V-D-J combinations in PD-1$^{+/-}$ mice among the pre-selection total TCRβ repertoire, pre-selection unique TCRβ repertoire, post-selection total TCRβ repertoire, and post-selection unique TCRβ repertoire (**E**). The number of differential V-D-J combinations that were unique to each group was shown in the non-overlapping sections, and the number of differential V-D-J combinations that was common to two, three, and all four groups was indicated in the relevant overlapping areas. Data were derived from n = 3 biological replicates per group.

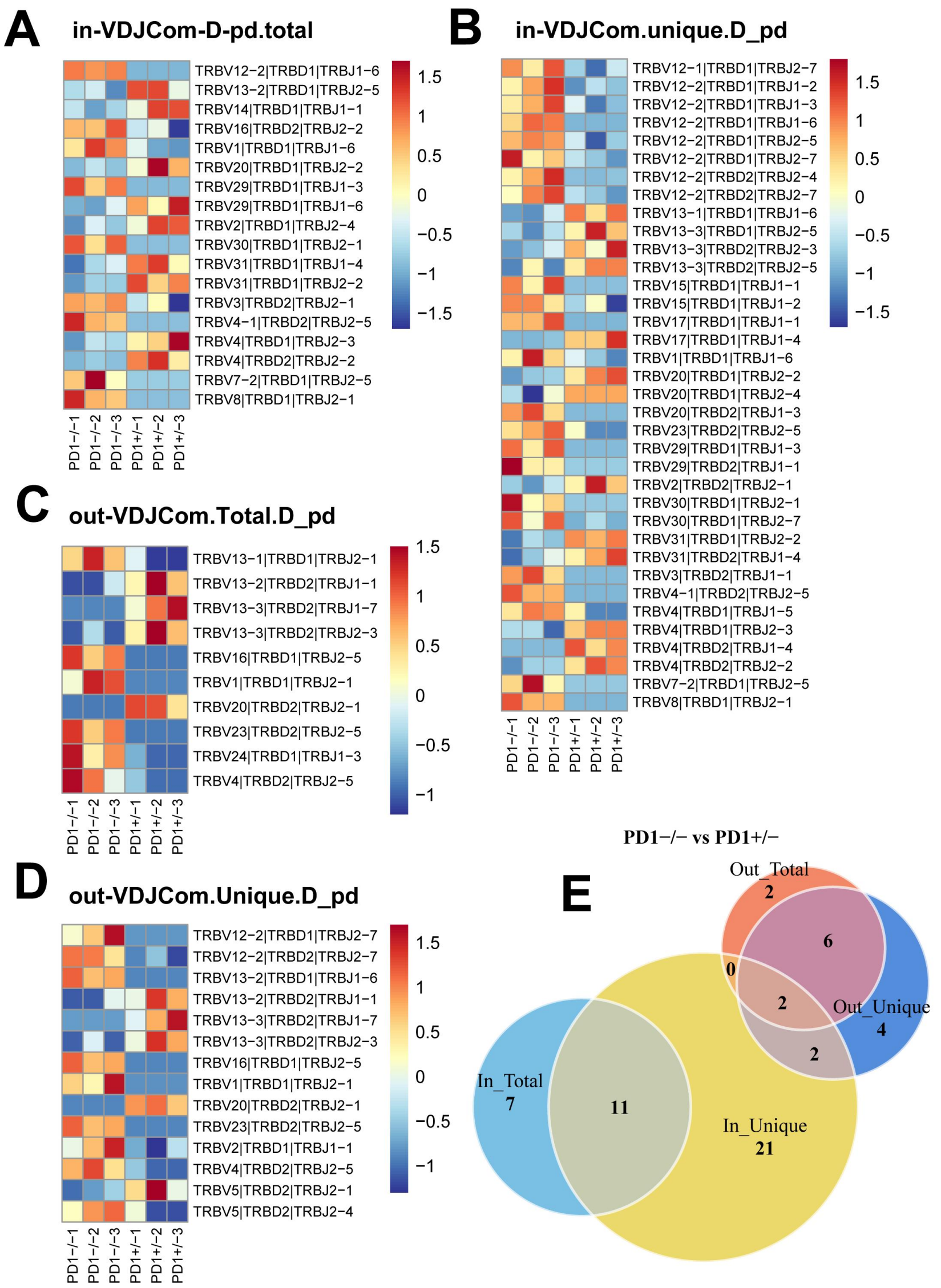

Figure S4. Differential usage frequencies of V-D-J combinations between PD-1$^{-/-}$ mice and PD-1$^{+/-}$ mice. **A-D** Heatmaps showing differential V-D-J combinations between PD-1$^{-/-}$ mice and PD-1$^{+/-}$ mice in post-selection total TCRβ repertoire (**A**), post-selection unique TCRβ repertoire (**B**), pre-selection total TCRβ repertoire (**C**), pre-selection unique TCRβ repertoire (**D**). A Venn diagram showing the overlap degree of the differentially V-D-J combinations between PD-1$^{-/-}$ mice and PD -1$^{+/-}$ mice among the pre-selection total TCRβ repertoire, pre-selection unique TCRβ repertoire, post-selection total TCRβ repertoire, and post-selection unique TCRβ repertoire (**E**). The number of differential V-D-J combinations that were unique to each group was shown in the non-overlapping sections, and the number of differential V-D-J combinations that was common to two, three, and all four groups was indicated in the relevant overlapping areas. Data were derived from n = 3 biological replicates per group.

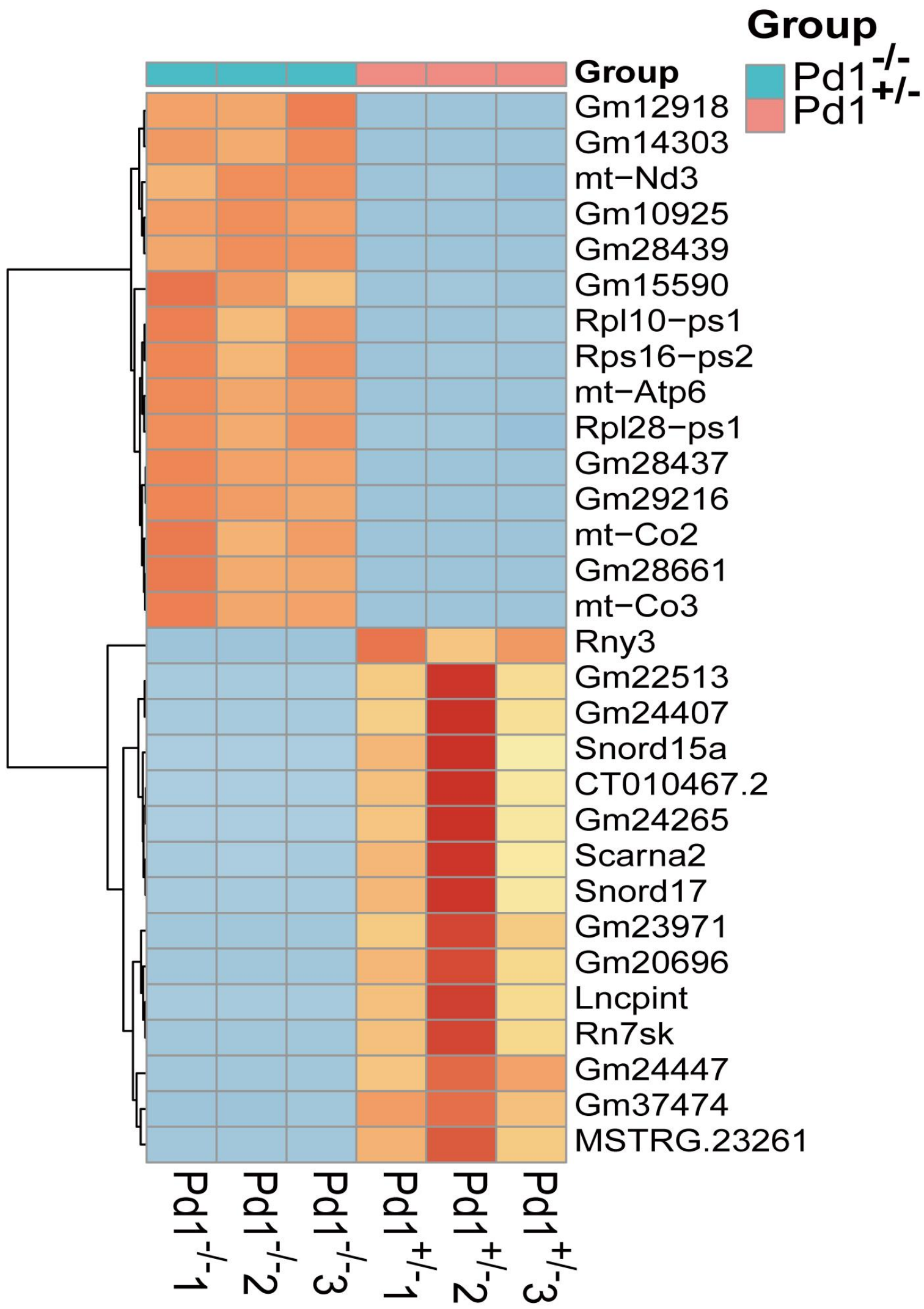

Figure S5. Heatmaps showing the differentially expressed genes (DEGs) between PD-$1^{-/-}$ mice group and PD-$1^{+/-}$ mice groups. Transcriptomic data were derived from n = 3 biological replicates per group. DEGs were identified using DESeq2 based on a threshold of |log2(Fold Change)| > 1.0 and adjusted $p < 0.05$.

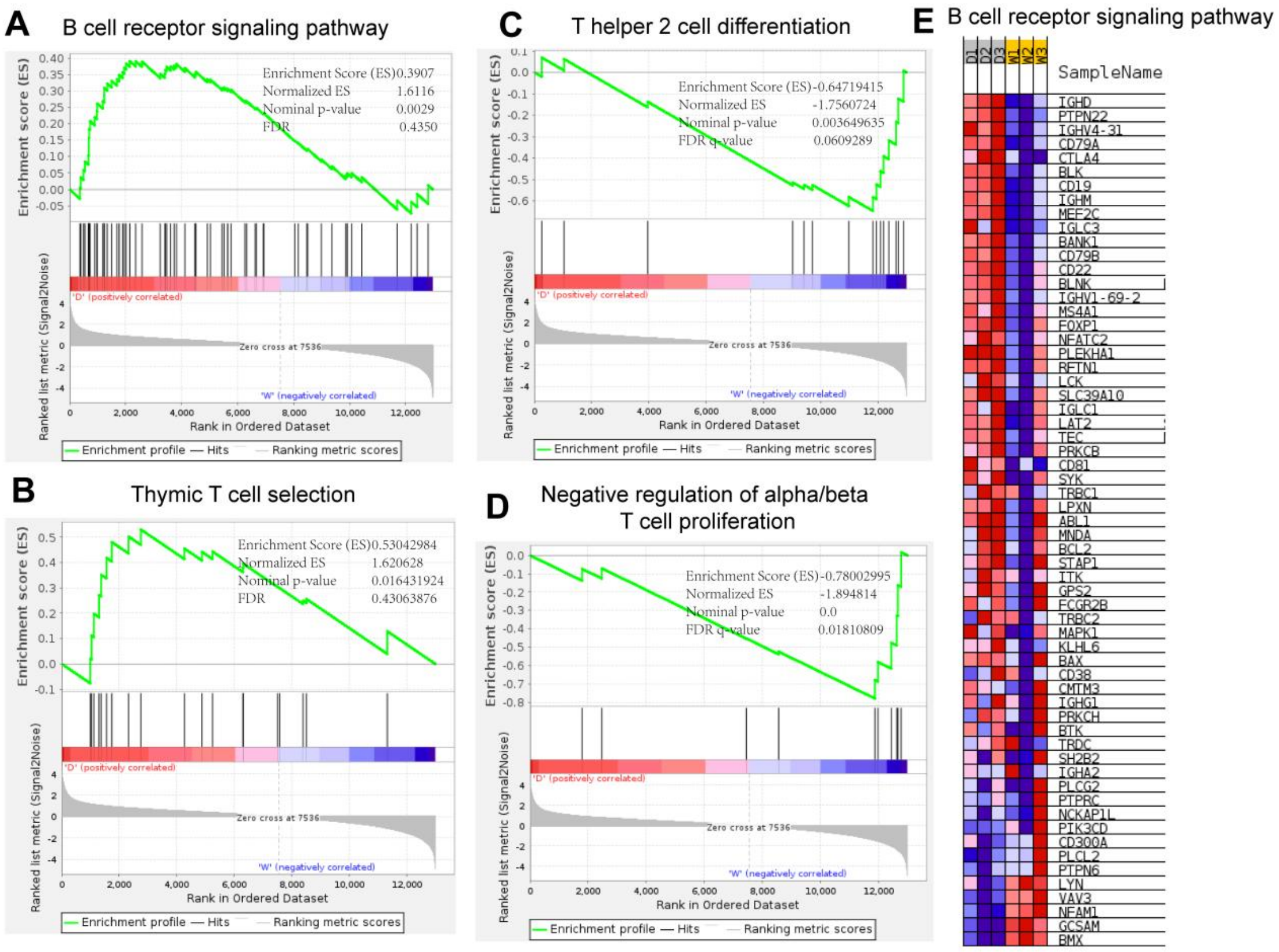

Figure S6. Gene Set Enrichment Analysis (GSEA) reveals altered signaling pathways in PD-1$^{-/-}$ mice. **A-D** GSEA plots showing enrichment of B cell receptor signaling pathway (**A**), thymic T cell selection (**B**), T helper 2 cell differentiation (**C**), and negative regulation of alpha/beta T cell proliferation (**D**) were notably changed in PD-1$^{-/-}$ mice. **E** Heatmaps showing the expression levels of genes involved in B cell receptor signaling pathway between PD-1$^{-/-}$ and WT groups. Transcriptomic data were derived from n = 3 biological replicates per group. DEGs were identified using DESeq2 based on a threshold of |log2(Fold Change)| > 1.0 and adjusted $p < 0.05$.

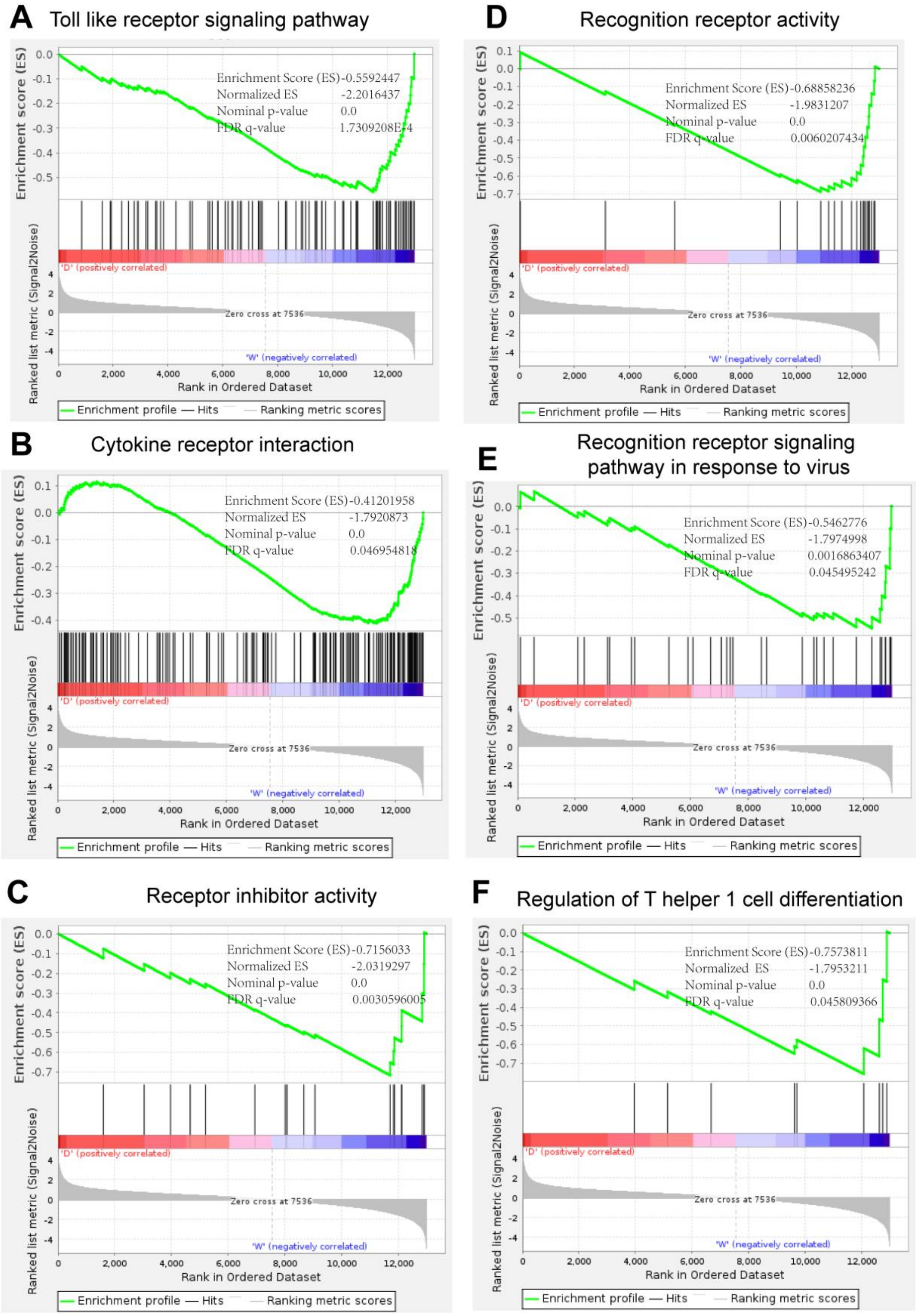

Figure S7. Gene Set Enrichment Analysis (GSEA) showing additional altered signaling pathways in PD-1$^{-/-}$ mice. **A-F** GSEA plots illustrating the enrichment of the Toll-like receptor signaling pathway (**A**), cytokine-cytokine receptor interaction (**B**), receptor inhibitor activity (**C**), recognition receptor activity (**D**), recognition receptor signaling pathway in response to virus (**E**), and regulation of T helper 1 cell differentiation (**F**) illustrating the enrichment of in PD-1$^{-/-}$ mice. Transcriptomic data were derived from n = 3 biological replicates per group.

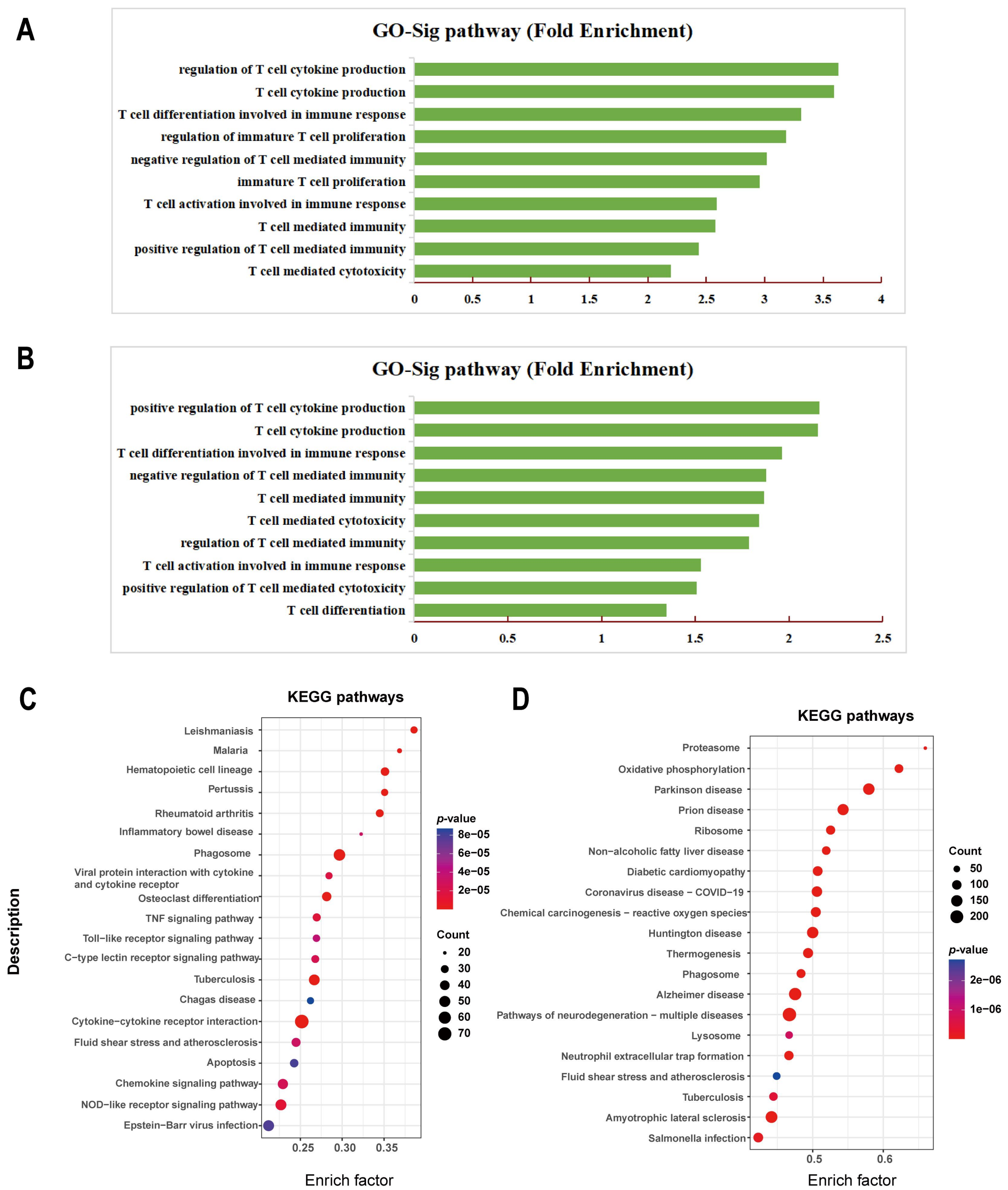

Figure S8. Gene Ontology (GO) and Kyoto Encyclopedia of Genes and Genomes (KEGG) enrichment analyses of differentially expressed genes (DEGs) in PD-1 gene-deficient mice. **A**, **B** GO enrichment analyses of the DEGs between PD-1$^{-/-}$ and WT mice (**A**) and between PD-1$^{+/-}$ and WT mice (**B**), the bar plot display the top 10 significantly enriched GO terms. **C**, **D** KEGG pathway enrichment analyses of the DEGs between PD-1$^{-/-}$ and WT mice (**C**), between PD-1$^{+/-}$ and WT mice (**D**), the bubble plot dispaly the top 20 significantly enriched KEGG pathway. Transcriptomic data were derived from n = 3 biological replicates per group.

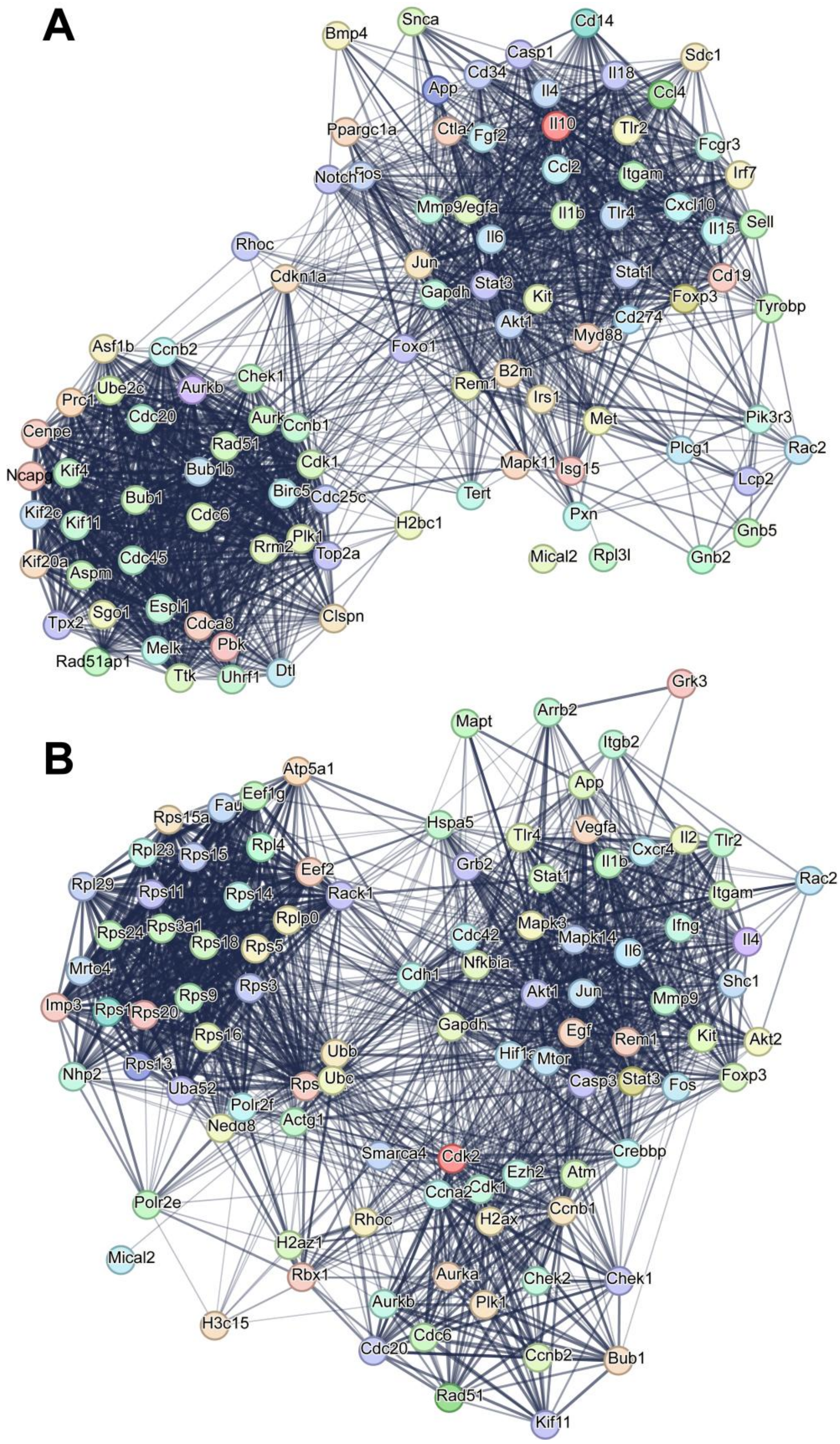

Figure S9. PPI networks generated from the top 100 DEGs between PD-1$^{-/-}$ and WT mice(**A**), and between PD-1$^{+/-}$and WT mice (**B**). Transcriptomic data were derived from n = 3 biological replicates per group.

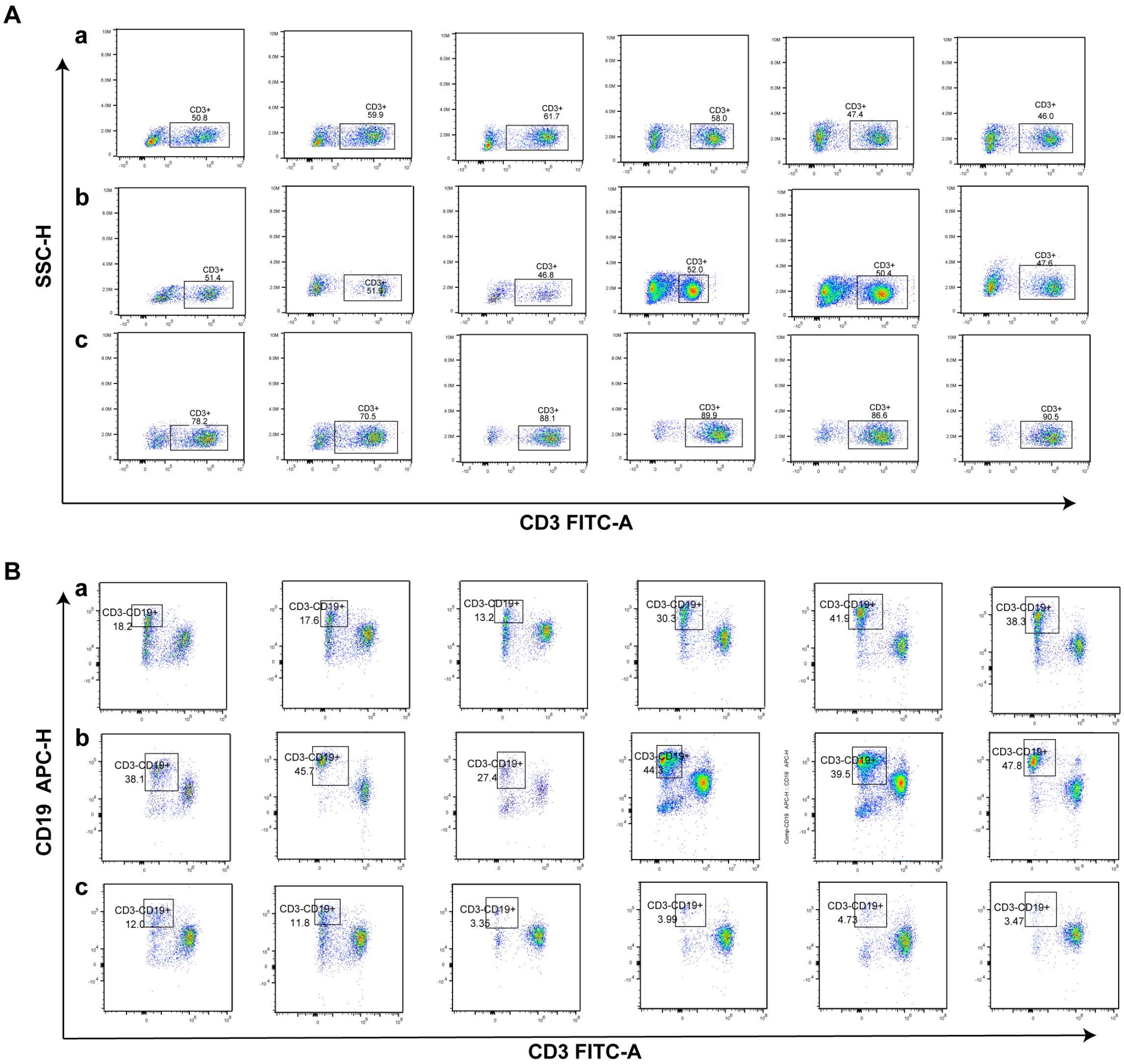

Figure S10. Representative flow cytometry plots of T-cell and B-cell populations. (**A**) Representative flow cytometry plots showing T cells populations in PD-1$^{+/-}$ (a) and WT (b), and PD-1$^{-/-}$ mice (c). (**B**) Representative flow cytometry plots showing B cells populations in PD-1$^{+/-}$ (a) and WT (b), and PD-1$^{-/-}$ mice (c). Flow cytometry data were derived from n = 6 biological replicates per group (3 females, 3 males). Statistical significance was determined using one-way ANOVA.

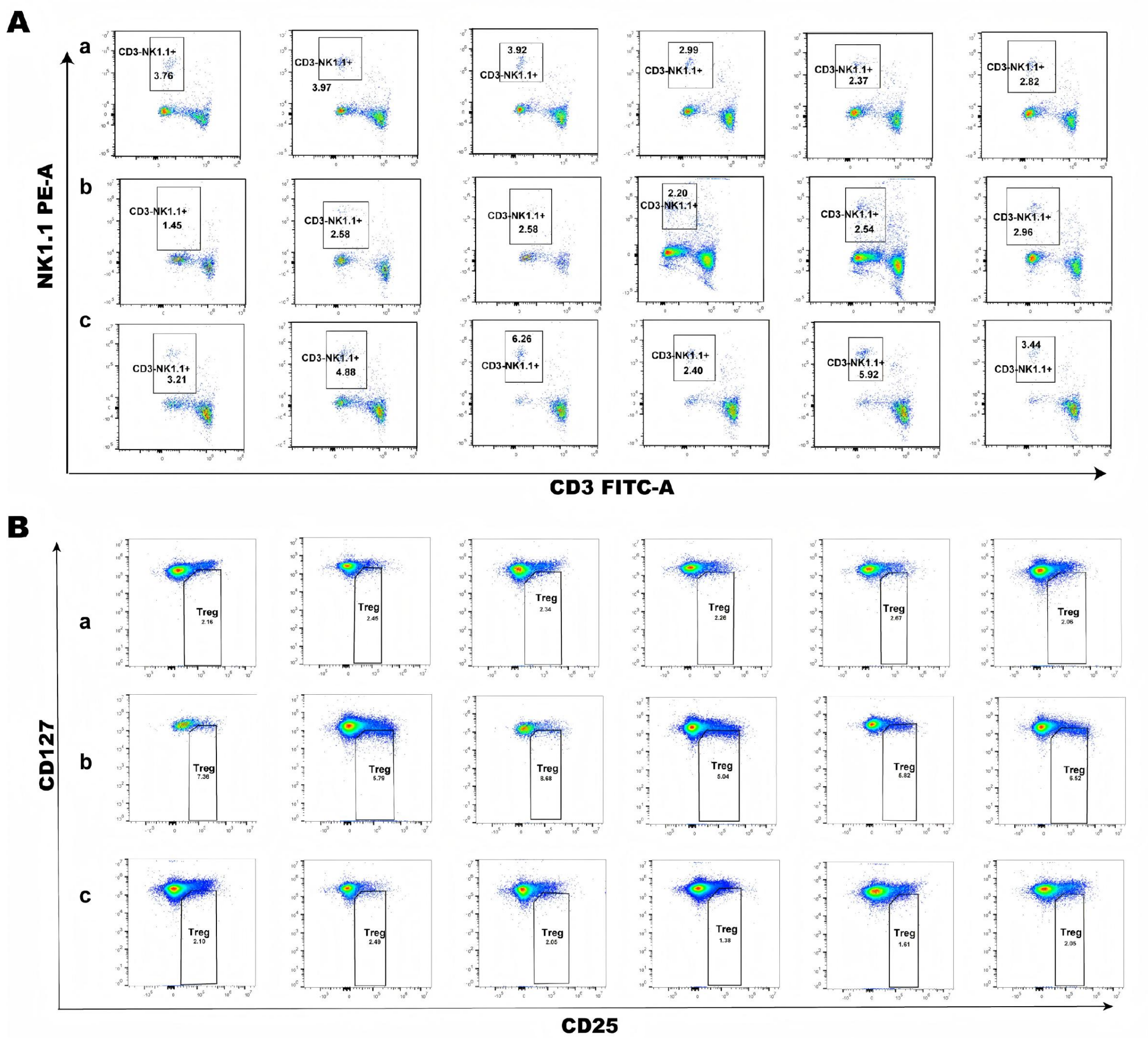

Figure S11. Representative flow cytometry plots of NK-cell and putative regulatory T-cell populations. (**A**) Representative flow cytometry plots showing NK cells populations in PD-$1^{+/-}$ (a) and WT (b), and PD-$1^{-/-}$ mice (c). (**B**) Representative flow cytometry plots showing the $CD4^{+}CD25^{+}CD127^{low/-}$ putative regulatory T-cell population in PD-$1^{+/-}$ (a) and WT (b), and PD-$1^{-/-}$ mice (c). The putative regulatory T-cell population was defined as $CD3^{+}$ $CD4^{+}$ $CD25^{+}$ $CD127^{low/-}$ cells. Flow cytometry data were derived from n = 6 biological replicates per group (3 females, 3 males). Statistical significance was determined using one-way ANOVA.

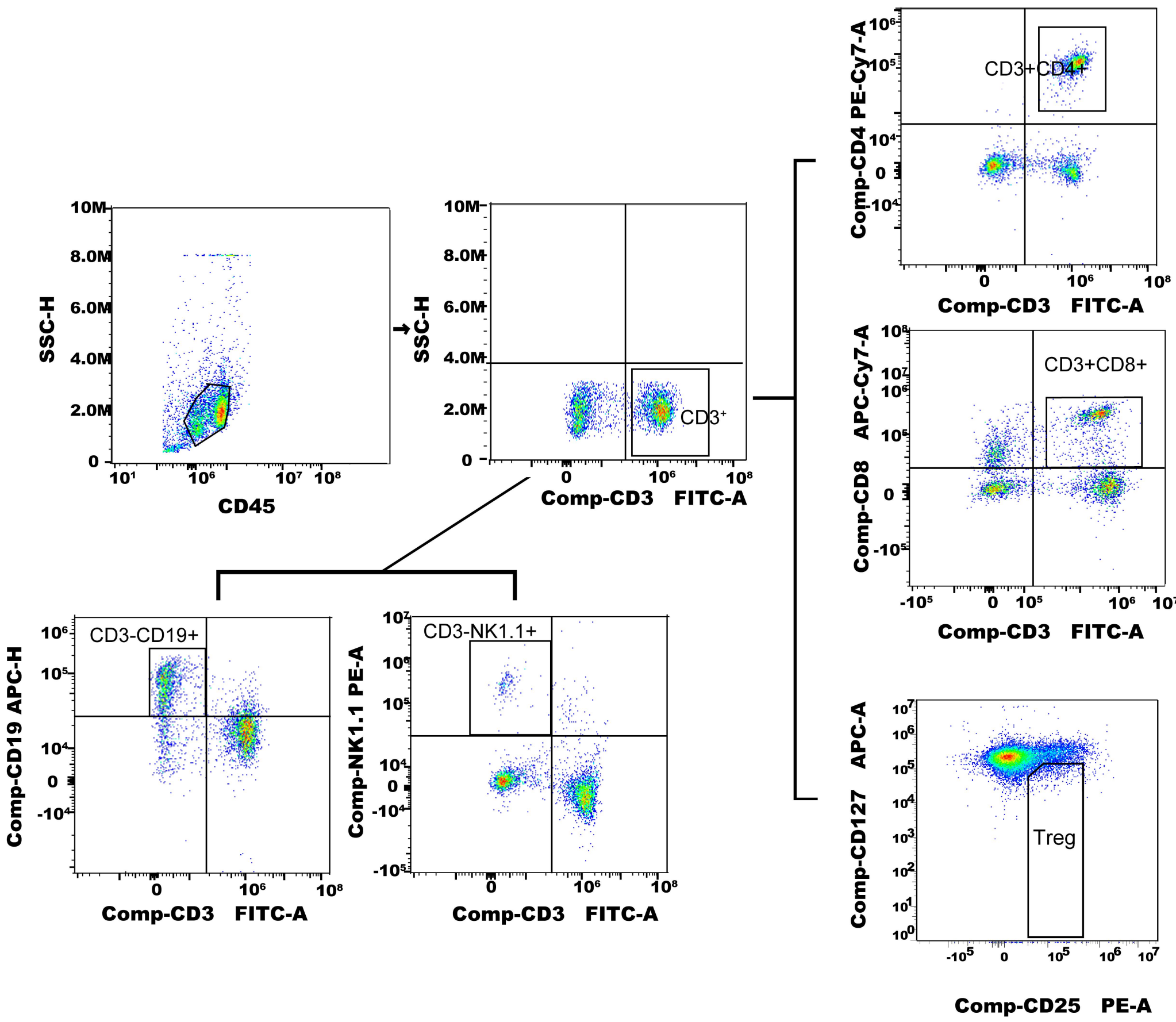

Figure S12. Representative flow cytometry gating strategy for immune cell subset identification. Representative flow cytometry plots illustrating sequential gating for lymphocyte identification based on forward and side scatter characteristics, doublet exclusion, and the subsequent identification of T lymphocytes ($CD3^+$), B lymphocytes ($CD19^+$), natural killer cells ($CD3^-$ $NK1.1^+$), $CD4^+$ and $CD8^+$ T-cell subsets, and the $CD4^+CD25^+CD127^{low/-}$ putative regulatory T-cell population.

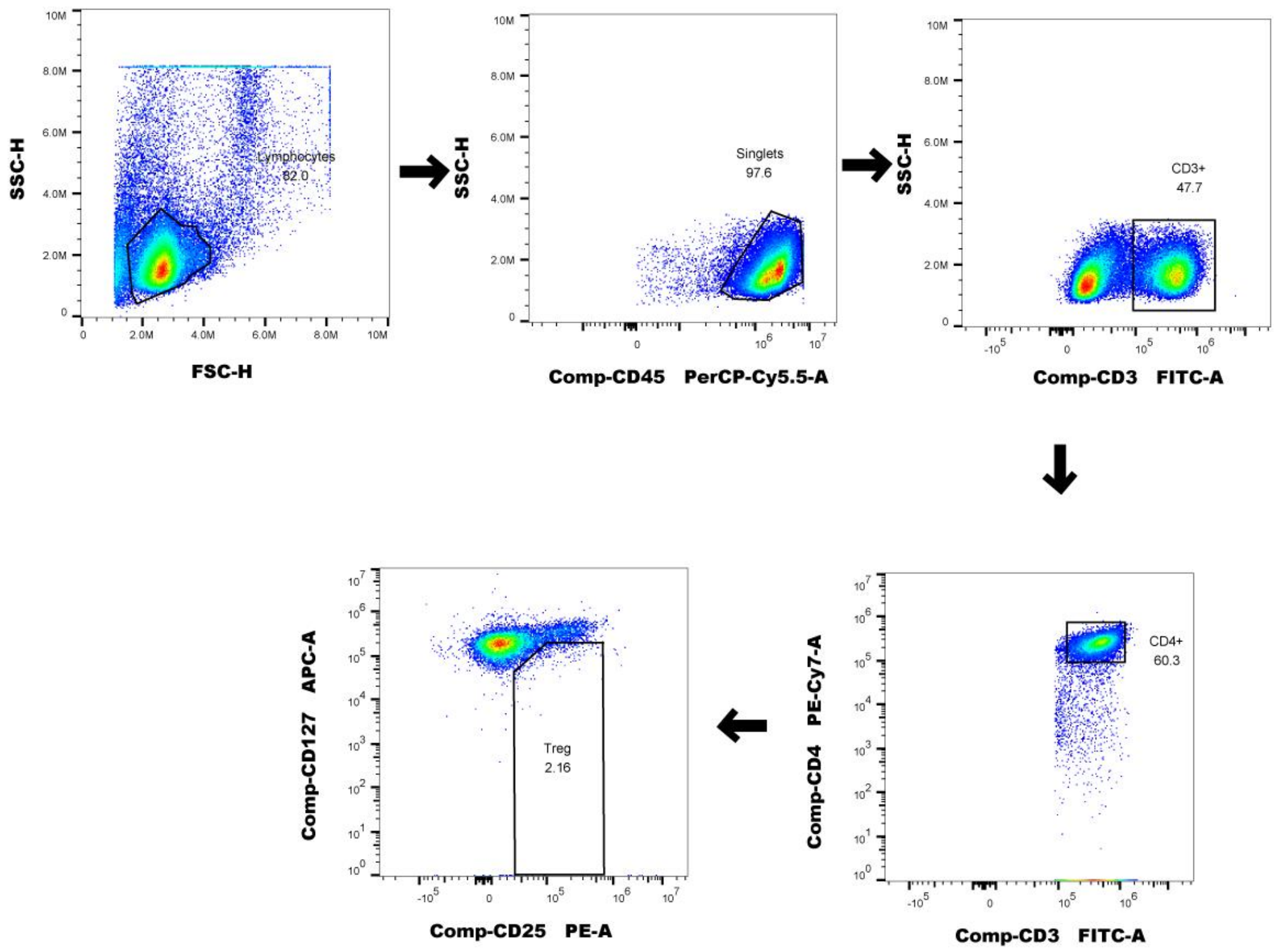

Figure S13. Comprehensive sequential flow cytometry gating strategy for identifying the CD4$^+$CD25$^+$CD127$^{low/-}$ putative regulatory T-cell population. This figure illustrates the sequential gating approach utilized to isolate the CD4$^+$CD25$^+$CD127$^{low/-}$ regulatory T cell population. Representative flow cytometry plots are derived from the splenic suspension of a PD-1$^{+/-}$ heterozygous mouse. The plots delineate the initial selection of the lymphocyte population, the subsequent exclusion of doublets to ensure singlet analysis, and the downstream identification of CD3$^+$ T cells and the CD4$^+$ T-cell subset. The final panel illustrates the gated CD4$^+$CD25$^+$CD127$^{low/-}$ putative regulatory T-cell population within the CD3$^+$CD4$^+$ T-cell compartment.
