# Supplementary material for: PD-1 deficiency reshapes the TCRβ repertoire and T-cell developmental programs associated with susceptibility to lupus-like autoimmunity
Source: Front Immunol. 2026 Sep 15;17:1911666. doi: 10.3389/fimmu.2026.1911666 (PMC13619840; doi:10.3389/fimmu.2026.1911666)
Supplement: Supplementary file 2 [file Table1.doc]

**Table S1**. TCRβ CDR3 sequence statistics

| ID | Read Pair | Filter reads | Filter rate（%） | Clones | Unique  CDR3aa | Unique  CDR3nt | Out of frame clones(%) |
| --- | --- | --- | --- | --- | --- | --- | --- |
| Pd1^-/-^-1 | 10344086 | 9831098 | 4.96 | 11612 | 2608 | 2596 | 6.61 |
| Pd1^-/-^-2 | 10114465 | 9506940 | 6.01 | 17815 | 3707 | 3682 | 6.38 |
| Pd1^-/-^-3 | 11073418 | 10389843 | 6.17 | 19740 | 3350 | 3337 | 4.64 |
| Pd1^+/-^-1 | 8354301 | 7923910 | 5.15 | 11264 | 3883 | 3862 | 5.32 |
| Pd1^+/-^-2 | 9016206 | 8567385 | 4.98 | 6859 | 1902 | 1889 | 4.47 |
| Pd1^+/-^-3 | 10875423 | 10306913 | 5.23 | 6085 | 1399 | 1393 | 7.61 |
| WT-1 | 10862938 | 10233119 | 5.80 | 12366 | 2196 | 2182 | 7.74 |
| WT-2 | 11208226 | 10470353 | 6.58 | 11083 | 1761 | 1747 | 7.14 |
| WT-3 | 11571928 | 10897149 | 5.83 | 15433 | 2308 | 2294 | 6.48 |
